# Intercornual distance and postoperative reproductive outcomes in moderate-to-severe intrauterine adhesions: a retrospective cohort study

**DOI:** 10.1093/hropen/hoag038

**Published:** 2026-05-05

**Authors:** Yifu He, Xingping Zhao, Huan Huang, Changfa Shu, Xiaoming Guan, Jia Guo, Dabao Xu

**Affiliations:** Department of Obstetrics and Gynecology, The Third Xiangya Hospital, Central South University, Changsha, China; Branch of National Clinical Research Center for Obstetrics and Gynecology, The Third Xiangya Hospital, Central South University, Changsha, China; Center for Gynecological Disease and Reproductive Health, Furong Laboratory, Changsha, China; Engineering Research Center for Intelligent Equipment and Products in the Diagnosis and Treatment of Gynecological Diseases, Changsha, China; Innovation Center for Hysteroscopic Cold Scissor Technique, Changsha, China; Department of Obstetrics and Gynecology, The Third Xiangya Hospital, Central South University, Changsha, China; Branch of National Clinical Research Center for Obstetrics and Gynecology, The Third Xiangya Hospital, Central South University, Changsha, China; Center for Gynecological Disease and Reproductive Health, Furong Laboratory, Changsha, China; Engineering Research Center for Intelligent Equipment and Products in the Diagnosis and Treatment of Gynecological Diseases, Changsha, China; Innovation Center for Hysteroscopic Cold Scissor Technique, Changsha, China; Department of Obstetrics and Gynecology, The Third Xiangya Hospital, Central South University, Changsha, China; Branch of National Clinical Research Center for Obstetrics and Gynecology, The Third Xiangya Hospital, Central South University, Changsha, China; Center for Gynecological Disease and Reproductive Health, Furong Laboratory, Changsha, China; Engineering Research Center for Intelligent Equipment and Products in the Diagnosis and Treatment of Gynecological Diseases, Changsha, China; Innovation Center for Hysteroscopic Cold Scissor Technique, Changsha, China; Department of Obstetrics and Gynecology, The Third Xiangya Hospital, Central South University, Changsha, China; Branch of National Clinical Research Center for Obstetrics and Gynecology, The Third Xiangya Hospital, Central South University, Changsha, China; Center for Gynecological Disease and Reproductive Health, Furong Laboratory, Changsha, China; Engineering Research Center for Intelligent Equipment and Products in the Diagnosis and Treatment of Gynecological Diseases, Changsha, China; Innovation Center for Hysteroscopic Cold Scissor Technique, Changsha, China; Department of Obstetrics and Gynecology, Baylor College of Medicine, Houston, TX, USA; Xiangya School of Nursing, Central South University, Changsha, China; Department of Obstetrics and Gynecology, The Third Xiangya Hospital, Central South University, Changsha, China; Branch of National Clinical Research Center for Obstetrics and Gynecology, The Third Xiangya Hospital, Central South University, Changsha, China; Center for Gynecological Disease and Reproductive Health, Furong Laboratory, Changsha, China; Engineering Research Center for Intelligent Equipment and Products in the Diagnosis and Treatment of Gynecological Diseases, Changsha, China; Innovation Center for Hysteroscopic Cold Scissor Technique, Changsha, China

**Keywords:** intrauterine adhesions, intercornual distance, risk stratification, clinical pregnancy, hysteroscopy

## Abstract

**STUDY QUESTION:**

Does intercornual distance (ICD), as reflected by intrauterine stent size during hysteroscopic adhesiolysis, have an association with reproductive outcomes and provide information for postoperative risk stratification in women with moderate-to-severe intrauterine adhesions?

**SUMMARY ANSWER:**

A smaller ICD was independently associated with a lower likelihood of clinical pregnancy within 1 year of the final hysteroscopy and may offer additional information for postoperative risk stratification in women with moderate-to-severe intrauterine adhesions.

**WHAT IS KNOWN ALREADY:**

Reproductive outcomes following treatment for moderate-to-severe intrauterine adhesions vary considerably. Existing severity classifications offer limited discrimination within this group. The potential association between ICD and postoperative reproductive outcomes has not been systematically evaluated.

**STUDY DESIGN, SIZE, DURATION:**

Retrospective cohort study conducted at a tertiary academic center from November 2022 to October 2023. A total of 560 women were included, with up to 2 years of follow-up.

**PARTICIPANTS/MATERIALS, SETTING, METHODS:**

Retrospective single-center cohort of women aged 18–44 years with moderate-to-severe intrauterine adhesions (American Fertility Society [AFS] score ≥ 5) undergoing standardized hysteroscopic management with adhesiolysis, intrauterine stent placement, and scheduled second-look hysteroscopy. ICD was reflected by intraoperatively selected stent size, categorized into ordered levels (XXXS–XXXL) and dichotomized using an XS threshold (∼20–22 mm). Postoperative ultrasound-derived ICD was analyzed for supportive evaluation. The primary outcome was clinical pregnancy within 1 year of the final hysteroscopy. Associations were examined using multivariable logistic regression with sensitivity analyses. The incremental predictive value and clinical utility of stent-size classification (≤XS vs >XS) beyond established clinical variables were evaluated using discrimination, calibration, and decision-curve analyses.

**MAIN RESULTS AND THE ROLE OF CHANCE:**

Women in the ≤XS group had lower odds of clinical pregnancy within 1 year of the final hysteroscopy compared with those in the >XS group (adjusted odds ratio [aOR] = 0.447, 95% CI 0.255–0.786; *P* = 0.005). The association was consistent in sensitivity analyses and when postoperative ultrasound-derived ICD was analyzed as a continuous variable. Although improvements in model discrimination were modest, the difference in the probability of clinical pregnancy between the ≤XS and >XS groups was 21.2% (absolute risk difference [ARD] = 21.2%, 95% CI 9.6–32.2; *P* < 0.001), corresponding to a number needed to benefit (NNB) of 4.7, indicating potential clinical utility for postoperative risk stratification.

**LIMITATIONS, REASONS FOR CAUTION:**

This single-center retrospective study may limit generalizability and is subject to selection bias and residual confounding. Prospective multicenter validation is required.

**WIDER IMPLICATIONS OF THE FINDINGS:**

ICD may provide additional information for postoperative risk stratification in women with moderate-to-severe intrauterine adhesions.

**STUDY FUNDING/COMPETING INTEREST(S):**

This study was supported by Scientific and Technological Project of Furong Laboratory (Gynecological Disease and Reproductive Health) (No. 2023SK2109) and Key Research Project of National Key Clinical Specialties, Health Commission of Hunan Province (Z2023068), China. The authors have no conflicts of interest to declare.

**TRIAL REGISTRATION NUMBER:**

N/A.

WHAT DOES THIS MEAN FOR PATIENTS? Women with moderate-to-severe intrauterine adhesions (scar tissue inside the uterus) often undergo surgery with the expectation of increasing their chance of pregnancy. However, even after standardized treatment, pregnancy outcomes remain variable. In this study of more than 500 women, we found that intercornual distance (ICD), the distance between the upper corners of the uterine cavity, was related to the chance of pregnancy within 1 year after surgery. More specifically, women with a smaller ICD were less likely to conceive within the first postoperative year.

ICD can be estimated during surgery and assessed using ultrasound. Although it cannot precisely predict individual pregnancy outcomes, it may help to identify those who could benefit from closer follow-up or earlier discussions regarding additional fertility support. Thus, understanding ICD may support shared decision-making between patients and their doctors when planning subsequent fertility steps. Further studies are required before this approach can be integrated into routine clinical practice, but it may represent a step toward more personalized management after surgery for intrauterine adhesions.

## Introduction

Intrauterine adhesions (IUA) is an acquired condition characterized by fibrotic bands within the uterine cavity following endometrial injury, leading to structural distortion and impaired endometrial receptivity (Yu *et al.*, 2008). Although hysteroscopic adhesiolysis is the standard treatment for moderate-to-severe IUA and postoperative anti-adhesion measures help reduce recurrence, reproductive outcomes remain suboptimal for many patients (Hooker *et al.*, 2014; Mortimer *et al.*, 2024; Munro *et al.*, 2025). Given that adverse reproductive outcomes are more frequently observed in women with moderate-to-severe disease, improving pregnancy outcomes in this subgroup remains a major clinical priority.

The American Fertility Society (AFS) scoring system is widely used to assess disease severity and has demonstrated clinical value in distinguishing reproductive outcomes between mild and moderate-to-severe IUA (American Fertility Society, 1988; Cao *et al.*, 2021). However, clinical experience suggests that even women with comparable AFS scores within the moderate-to-severe category who undergo similar standardized management may still experience markedly different reproductive outcomes. To address this, intensified or adjunctive interventions—such as stem cell-based therapies, exosomes, and novel biomaterial scaffolds—have been explored (Li *et al.*, 2021; Cen *et al.*, 2022; Lee *et al.*, 2025). Nevertheless, these approaches are resource-intensive and not widely generalizable, underscoring the need to identify patients most likely to benefit from such strategies. At present, practical and reproducible parameters for postoperative stratification within the moderate-to-severe spectrum remain limited.

In an earlier exploratory cohort at our center, ultrasound-derived intercornual distance (ICD) appeared to be associated with reproductive outcomes in women with moderate-to-severe IUA (Lei *et al.*, 2022). However, this observation has not been systematically evaluated in the context of reproductive outcomes.

Within our standardized hysteroscopic management protocol, an intrauterine stent is routinely placed to minimize postoperative adhesion recurrence, with stent size selected based on ICD. Specifically, stent size is determined by fixed physical dimensions, and the stent is positioned to achieve fundal contact with a relatively consistent clearance from the lateral uterine walls, thereby providing a practical approximation of ICD under hysteroscopic visualization. The present study therefore aimed to systematically evaluate the association between ICD as reflected by stent size and reproductive outcomes. In addition, postoperative ultrasound-derived ICD was analyzed to assess the agreement between the two approaches and to explore its association with reproductive outcomes.

## Materials and methods

### Study design

This single-center retrospective cohort study was conducted at the Third Xiangya Hospital of Central South University. Clinical data were retrospectively extracted from the hospital’s electronic medical record system for consecutive patients who initiated a standardized hysteroscopic treatment protocol between 1 November 2022 and 31 October 2023.

### Ethics and registration

Ethical approval was obtained from the Ethics Committee of the Third Xiangya Hospital, Central South University (No. 241053). The study adhered to the STROCSS 2021 guidelines. Owing to its anonymized and retrospective design, the requirement for written informed consent was waived.

### Study population

Women were eligible for inclusion if they met the following criteria: (i) age 18–44 years; (ii) a diagnosis of moderate-to-severe IUA, defined according to the AFS classification (score ≥ 5); (iii) treatment within the institutional standardized hysteroscopic management pathway, consisting of initial adhesiolysis with intrauterine stent placement followed by a planned second-look procedure, with stent removal performed prior to hysteroscopic cavity reassessment and repeat adhesiolysis when necessary; and (iv) documented intention to conceive after surgery with no contraindications to pregnancy based on reproductive evaluation.

Patients were excluded if they had (i) congenital genital tract malformations or (ii) missing essential reproductive information (including mode of conception or documented postoperative reproductive plan) that could not be retrieved from medical records or follow-up, thereby precluding determination of mode of conception.

### Surgical procedure

All procedures were performed by the same senior surgeon with a consistent surgical team. Patients underwent an institutional standardized hysteroscopic management pathway consisting of adhesiolysis with intrauterine stent (KMS Medical Technology Co., Ltd., Changsha, China) placement followed by scheduled second-look hysteroscopy (technical details are provided in the Supplementary Materials and Methods). Adhesiolysis was continued until restoration of the uterine cavity architecture with clear visualization of both tubal ostia, which served as the predefined criterion for satisfactory reconstruction prior to stent placement.

Intrauterine stents were routinely placed to reduce postoperative adhesion recurrence (Supplementary Fig. S1). Stent size selection was primarily guided by preoperative ultrasound-derived ICD, with final determination based on intraoperative hysteroscopic assessment of the uterine cavity. Uterine depth was measured to ensure appropriate stent placement and adequate fundal reach, and final positioning was confirmed hysteroscopically. Optimal placement was defined as full fundal contact with symmetrical lateral expansion, with the lateral margins positioned approximately one 5-Fr operative instrument width (≈1.5–2 mm) from the lateral uterine wall, ensuring adequate coverage without excessive compression (Supplementary Fig. S2).

Second-look hysteroscopy was generally recommended after two to three menstrual cycles, during the early proliferative phase (cycle days 3–7), although the timing could be adjusted based on individual clinical circumstances. The actual interval between procedures was recorded and included as a covariate in multivariable analyses. At the second-look procedure, the intrauterine stent was first removed via the retrieval tail positioned within the cervical canal, without cervical dilation or hysteroscopic guidance. Hysteroscopic reassessment of the uterine cavity was then performed. After reassessment, patients were advised that attempts to conceive could be initiated based on clinical evaluation and reproductive planning.

### Ultrasound assessment

Three-dimensional transvaginal ultrasound (3D-TVS) examinations performed before and after surgery were retrieved from the institutional electronic medical record system. All examinations were performed using Voluson E8 and Voluson E10 ultrasound systems (GE Healthcare, Chicago, IL, USA) at the same center, following a standardized acquisition protocol with 3D coronal reconstruction. For each patient, the ultrasound examinations closest in time to the initial hysteroscopic procedure (i.e. the stent placement procedure) were identified, and the corresponding ultrasound-derived ICD values were extracted. ICD was defined as the linear distance between the bilateral uterine cornua at the junction of the endometrial cavity and the tubal ostia (Supplementary Fig. S3). Detailed ultrasound acquisition and measurement procedures are described in the Supplementary Materials and Methods.

### Data collection and follow-up

Clinical information and reproductive outcome data were obtained from the hospital’s electronic medical record system and a standardized follow-up form. Follow-up was conducted by the same gynecologist via telephone using this form. Collected information included mode of conception (natural conception or ART), confirmation of pregnancy, pregnancy outcomes, and live birth details. For patients successfully contacted, the reported mode of conception was verified and found to be consistent with the postoperative reproductive plan documented in the medical records. For those who could not be reached, the postoperative reproductive plan recorded in the medical records was used to determine the mode of conception for analysis. All data were independently extracted and cross-checked by two senior gynecologists to ensure accuracy.

### Outcome measures

The primary outcome was clinical pregnancy within 1 year of the final hysteroscopy, defined as visualization of a gestational sac on ultrasound or histopathological confirmation of products of conception. Pregnancies were categorized by mode of conception as natural conception or ART. Secondary outcomes included two complementary live-birth endpoints: (i) live birth following the first clinical pregnancy within 1 year of the final hysteroscopy (delivery at ≥ 28 weeks with signs of life) and (ii) cumulative live birth within 2 years of the final hysteroscopy, defined as at least one live birth within this period. Because fertility treatments and obstetric management were not fully captured, live birth analyses were considered secondary and exploratory.

### Statistical analysis

Baseline demographic, clinical, and reproductive characteristics were summarized for the overall cohort and compared across the nine ordered stent-size categories (XXXS–XXXL) using one-way ANOVA or Kruskal–Wallis tests for continuous variables and Fisher’s exact test with Monte Carlo simulation for categorical variables. Linear trends across the ordered stent-size categories were evaluated using the linear-by-linear association test, with effect size expressed as Kendall’s τ-b.

To explore a potential prognostic threshold, an exploratory scan across the ordered stent-size categories was performed by evaluating each candidate cut-off (≤category vs >category). The XS category was selected as the candidate threshold based on these evaluations, and its stability was assessed using bootstrap resampling (B = 300). Based on this threshold, patients were subsequently classified according to stent-size classification (≤XS vs >XS) for downstream analyses. In these analyses, stent-size classification was used to represent relative differences in ICD, with ≤XS indicating smaller ICD and >XS indicating larger ICD. Additionally, agreement between the nine stent-size categories and postoperative ultrasound-derived ICD was assessed by comparing ultrasound-derived ICD with ICD ranges reflected by stent size and quantified using weighted Cohen’s kappa, with exact agreement and agreement within ±1 category defined *a priori*. Changes in ultrasound-derived ICD between preoperative and postoperative assessments were further examined among patients with both preoperative and postoperative ultrasound-derived ICD available.

Baseline demographic, clinical, and reproductive characteristics were compared between the ≤XS and >XS groups based on stent-size classification using the Student’s *t*-test or Mann–Whitney *U*-test for continuous variables and the χ^2^ test or Fisher’s exact test for categorical variables, as appropriate. Associations between stent-size classification (≤XS vs >XS) and clinical pregnancy within 1 year of the final hysteroscopy were assessed using logistic regression. Variables with *P* < 0.10 in univariable analyses and no evidence of multicollinearity (variance inflation factor < 5) were entered into multivariable models. Postoperative ultrasound-derived ICD was additionally analyzed as a continuous variable (per 1-mm increase). Subgroup analyses were conducted according to conception mode (natural vs ART). Propensity score-based sensitivity analyses included inverse probability of treatment weighting (IPTW) and 1:2 nearest-neighbor propensity score matching (PSM), with covariate balance assessed using standardized mean differences (|SMD| < 0.10). Secondary live birth outcomes were analyzed using analogous multivariable logistic models and interpreted as exploratory.

Time-to-pregnancy was analyzed using Kaplan–Meier curves, log-rank tests, and Cox proportional hazards regression. It was defined as the interval between the date of the final hysteroscopic procedure and the date of clinical pregnancy. Women who did not achieve clinical pregnancy during follow-up were censored at the date of last contact or at 365 days, whichever occurred first. The proportional hazards assumption was assessed using Schoenfeld residuals. Given potential violations from this assumption, these analyses were considered exploratory.

To evaluate incremental predictive value, discrimination was assessed by comparing the area under the receiver operating characteristic curve (AUC) using the DeLong test. Calibration was evaluated using bootstrap internal validation, and clinical utility was assessed using decision curve analysis (DCA), the absolute risk difference (ARD), and the corresponding number needed to benefit (NNB). Exploratory machine learning analyses using a random forest classifier were conducted to evaluate predictive performance and the relative importance of ICD compared with other covariates. All analyses were performed using SPSS Statistics version 31.0 (IBM Corp., Armonk, NY, USA) and R version 4.5.1 (R Foundation for Statistical Computing, Vienna, Austria), with two-sided *P* < 0.05 considered statistically significant.

## Results

### Baseline demographic, clinical, and reproductive characteristics

**Figure 1.**
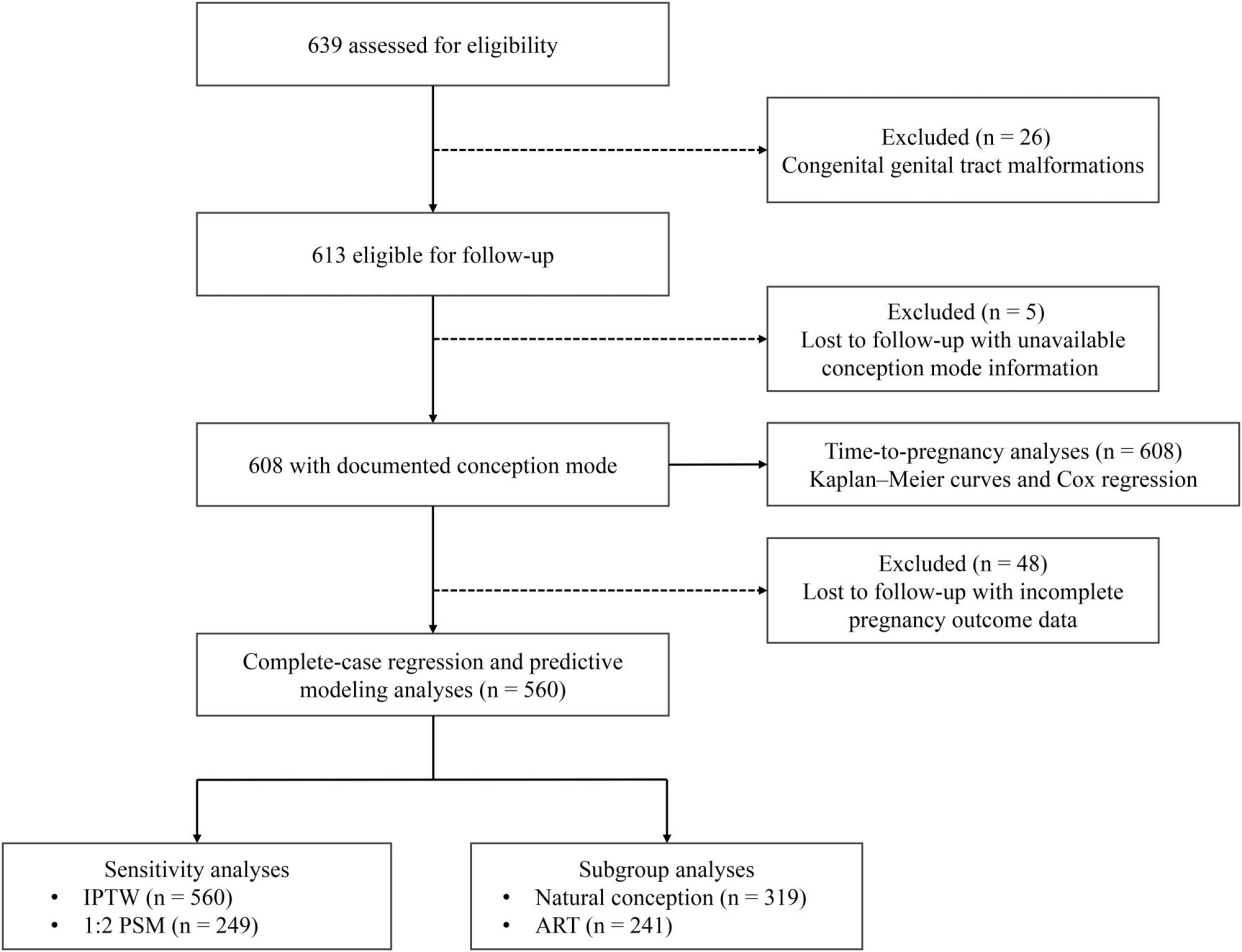
**Flow diagram of participant selection and analytic cohorts in women with moderate-to-severe intrauterine adhesions.** A total of 639 women with moderate-to-severe intrauterine adhesions (IUA) were assessed for eligibility. After excluding patients with congenital genital tract malformations (n = 26), 613 women were eligible for follow-up. Five women were excluded due to loss to follow-up and unavailable information on mode of conception. The remaining 608 women with documented mode of conception were included in time-to-pregnancy analyses using Kaplan–Meier curves and Cox proportional hazards regression. Among these 608 women, 48 were further excluded due to incomplete pregnancy outcome data during follow-up, leaving 560 women in the complete-case cohort for multivariable regression and predictive modeling analyses. Sensitivity analyses were conducted using IPTW (n = 560) and 1:2 PSM (n = 249). Subgroup analyses were performed according to mode of conception, including natural conception (n = 319) and ART (n = 241). IPTW, inverse probability of treatment weighting; IUA, intrauterine adhesions; PSM, propensity score matching.

Of 639 patients assessed, 26 were excluded due to congenital genital tract malformations, leaving 613 eligible for follow-up. Among these, five women could not be contacted and had no documented mode of conception in the medical records; they were therefore excluded from all analyses. The remaining 608 women had documented conception mode and were included in time-to-pregnancy analyses. Among these 608 women, 48 had incomplete pregnancy outcome data during follow-up and were excluded from complete-case regression analyses. Ultimately, 560 women comprised the primary analysis cohort, while 608 were included in time-to-pregnancy analyses (Fig. 1).

The characteristics of the 560 women are summarized in Table 1. Mean age was 33.7 ± 5.0 years and mean BMI was 21.7 ± 2.7 kg/m^2^. A scarred uterus was present in 25.2% of women, and 42.9% had undergone prior adhesiolysis. The median AFS score at the first hysteroscopic procedure was 8 (interquartile range [IQR] 8–10), improving to 3 (IQR 2–3) at second-look hysteroscopy; the distribution of initial AFS scores is presented in Supplementary Table S1. Mode of conception was natural conception in 57.0% and IVF in 43.0%. Overall, 61.4% achieved clinical pregnancy within 1 year of the final hysteroscopy (Table 1).

**Table 1.** Baseline characteristics, treatment-related variables, and reproductive outcomes according to stent-size classification (≤XS vs >XS).

Variable	Total (N = 560)	≤XS (N = 83)	>XS (N = 477)	*P*
**Baseline characteristics**				
Age, years	33.7 ± 5.0	33.3 ± 4.6	33.8 ± 5.1	0.433
BMI, kg/m²	21.7 ± 2.7	21.7 ± 2.8	21.7 ± 2.6	0.878
Ethnicity, n (%)				0.773
Han Chinese	501 (89.5%)	75 (90.4%)	426 (89.3%)	
Ethnic minorities	59 (10.5%)	8 (9.6%)	51 (10.7%)	
Menstrual cycle regularity, n (%)				0.023
Regular	472 (84.3%)	63 (75.9%)	409 (85.7%)	
Irregular	88 (15.7%)	20 (24.1%)	68 (14.3%)	
Menstrual duration, n (%)				0.337
Regular	524 (93.6%)	80 (96.4%)	444 (93.1%)	
Irregular	36 (6.4%)	3 (3.6%)	33 (6.9%)	
**Past gynecological history**				
Gravidity, n (%)				0.839
0	10 (1.8%)	2 (2.4%)	8 (1.7%)	
1–2	222 (39.6%)	33 (39.8%)	189 (39.6%)	
≥3	328 (58.6%)	48 (57.8%)	280 (58.7%)	
Parity, n (%)				0.068
0	280 (50.0%)	48 (57.8%)	232 (48.6%)	
1	194 (34.6%)	29 (34.9%)	165 (34.6%)	
≥2	86 (15.4%)	6 (7.2%)	80 (16.8%)	
Abortion, n (%)				0.150
0	23 (4.1%)	5 (6.0%)	18 (3.8%)	
1–2	347 (62.0%)	44 (53.0%)	303 (63.5%)	
≥3	190 (33.9%)	34 (41.0%)	156 (32.7%)	
Induced abortion, n (%)				0.793
0	221 (39.5%)	33 (39.8%)	188 (39.4%)	
1	171 (30.5%)	23 (27.7%)	148 (31.0%)	
≥2	168 (30.0%)	27 (32.5%)	141 (29.6%)	
Missed abortion, n (%)				0.766
0	263 (47.0%)	39 (47.0%)	224 (47.0%)	
1	190 (33.9%)	26 (31.3%)	164 (34.4%)	
≥2	107 (19.1%)	18 (21.7%)	89 (18.7%)	
Ectopic pregnancy, n (%)				0.949
No	487 (87.0%)	72 (86.7%)	415 (87.0%)	
Yes	73 (13.0%)	11 (13.3%)	62 (13.0%)	
Biochemical pregnancy, n (%)				0.652
No	498 (88.9%)	75 (90.4%)	423 (88.7%)	
Yes	62 (11.1%)	8 (9.6%)	54 (11.3%)	
Adenomyosis, n (%)				0.332
No	409 (73.0%)	57 (68.7%)	352 (73.8%)	
Yes	151 (27.0%)	26 (31.3%)	125 (26.2%)	
Scarred uterus due to prior uterine surgery, n (%)				0.180
No	419 (74.8%)	67 (80.7%)	352 (73.8%)	
Yes	141 (25.2%)	16 (19.3%)	125 (26.2%)	
Previous hysteroscopic adhesiolysis, n (%)				<0.001
No	320 (57.1%)	21 (25.3%)	299 (62.7%)	
Yes	240 (42.9%)	62 (74.7%)	178 (37.3%)	
**Treatment-related variables**				
Initial AFS score	8 (8–10)	10 (8–10)	8 (8–10)	0.007
Initial AFS severity grade, n (%)				0.004
Moderate (5–8)	317 (56.6%)	35 (42.2%)	282 (59.1%)	
Severe (9–12)	243 (43.4%)	48 (57.8%)	195 (40.9%)	
Initial DEGO grade, n (%)				<0.001
Grade 1	210 (37.5%)	8 (9.6%)	202 (42.3%)	
Grade 2	253 (45.2%)	38 (45.8%)	215 (45.1%)	
Grade 3	97 (17.3%)	37 (44.6%)	60 (12.6%)	
Stent placement duration, days	63 (56–80)	70 (59–91)	62 (55–78)	<0.001
Second AFS score	3 (2–3)	3 (2–4)	2 (2–3)	<0.001
Second AFS severity grade, n (%)				<0.001
0	96 (17.1%)	7 (8.4%)	89 (18.7%)	
Mild (1–4)	443 (79.1%)	63 (75.9%)	380 (79.7%)	
Moderate (5–8)	21 (3.8%)	13 (15.7%)	8 (1.7%)	
Second DEGO grade, n (%)				<0.001
Grade 1	367 (65.5%)	29 (34.9%)	338 (70.9%)	
Grade 2	160 (28.6%)	34 (41.0%)	126 (26.4%)	
Grade 3	33 (5.9%)	20 (24.1%)	13 (2.7%)	
Mode of conception, n (%)				0.014
Natural conception	319 (57.0%)	37 (44.6%)	282 (59.1%)	
ART	241 (43.0%)	46 (55.4%)	195 (40.9%)	
**Reproductive outcomes**				
Clinical pregnancy (1 year), n (%)	344 (61.4%)	36 (43.4%)	308 (64.6%)	<0.001
Live birth (first pregnancy), n (%)	283 (50.5%)	28 (33.7%)	255 (53.5%)	<0.001
Cumulative live birth (2 years), n (%)	316 (56.4%)	35 (42.2%)	281 (58.9%)	0.005

Values are presented as mean ± SD or median (interquartile range, IQR) for continuous variables, and as number (percentage) for categorical variables. *P* values represent comparisons between the ≤XS and >XS groups. Continuous variables were compared using the *t*-test or Mann–Whitney *U*-test, as appropriate. Categorical variables were compared using the chi-square test or Fisher’s exact test. AFS score and grade were defined according to the American Fertility Society classification system for intrauterine adhesions. DEGO grade was defined according to previously published criteria (Zhao *et al*., 2021). Clinical pregnancy (1 year): clinical pregnancy within 1 year of the final hysteroscopy; Live birth (first pregnancy): live birth following the first clinical pregnancy within 1 year of the final hysteroscopy; Cumulative live birth (2 years): cumulative live birth within 2 years of the final hysteroscopy.

AFS, American Fertility Society; DEGO, density of endometrial glandular openings; IQR, interquartile range.

Nine stent sizes (XXXS–XXXL) were defined as ordered categories based on predefined physical dimensions, with corresponding ICD ranges for each stent size summarized in Supplementary Table S2. Significant differences across stent-size categories were observed for age, parity, scarred uterus, prior adhesiolysis, AFS score, DEGO grade, stent placement duration, and mode of conception (Supplementary Table S3). A significant linear trend was observed for clinical pregnancy (*P* for trend < 0.01), with pregnancy rates decreasing toward smaller stent-size categories (Supplementary Table S4).

### Identification of a prognostic threshold based on stent-size classification

**Figure 2.**
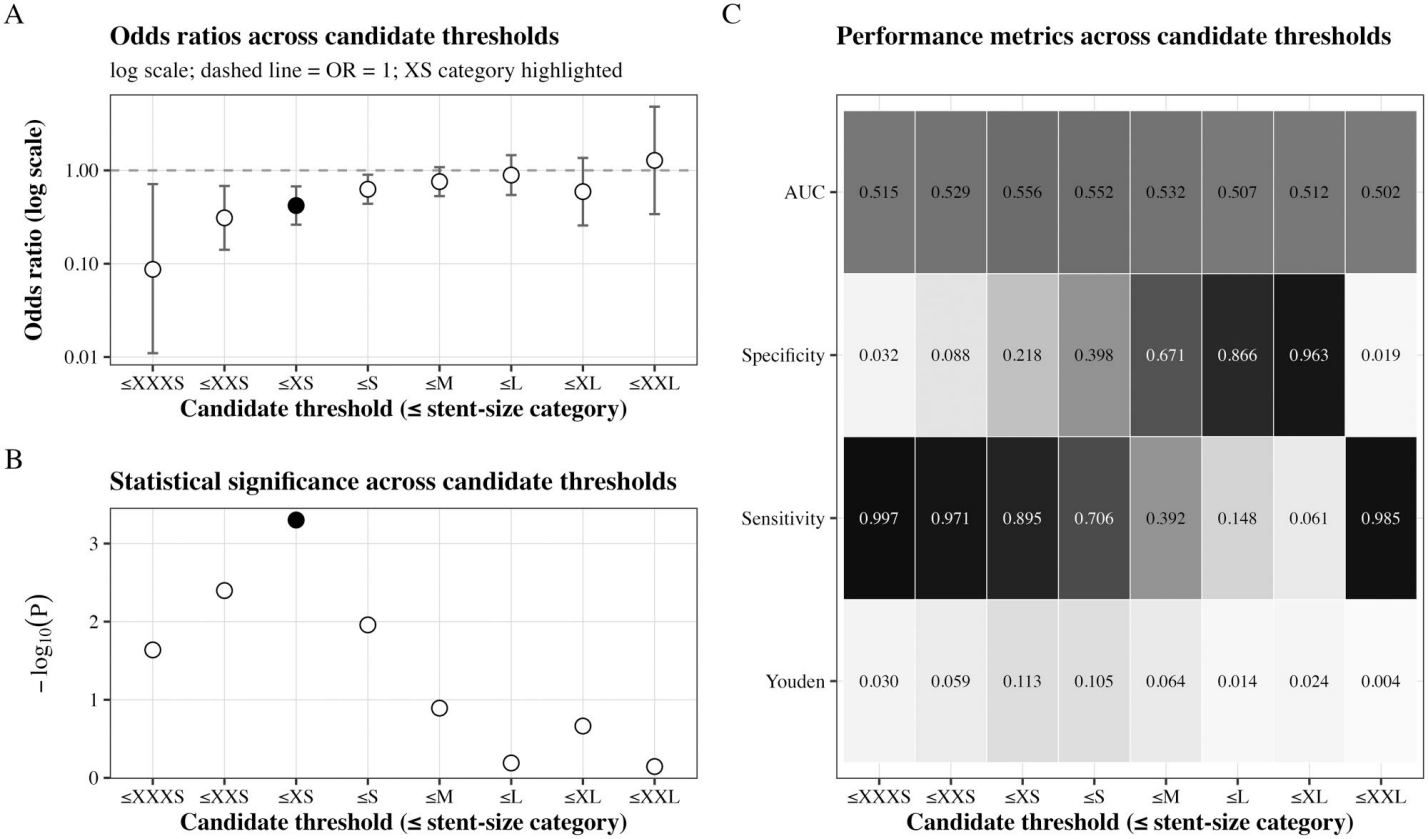
**Evaluation of candidate stent-size thresholds for predicting clinical pregnancy within 1 year of the final hysteroscopy.** (**A**) Odds ratios (ORs) for clinical pregnancy within 1 year of the final hysteroscopy across candidate thresholds based on stent-size categories, estimated using univariable logistic regression (log scale). The dashed horizontal line indicates OR = 1. The XS category is highlighted. (**B**) Statistical significance of associations across thresholds, expressed as −log10(*P*), derived from univariable logistic regression models. (**C**) Performance metrics across thresholds, including the Youden index, sensitivity, specificity, and area under the receiver operating characteristic curve (AUC). The XS category (corresponding to an intercornual distance of ∼20–22 mm) showed the highest discriminatory performance, with the highest AUC (0.556), greatest Youden index (0.113), and largest likelihood ratio χ^2^ (χ^2^ = 13.044; *P* < 0.001). This threshold was also associated with lower odds of clinical pregnancy (OR = 0.420, 95% CI 0.262–0.674; *P* < 0.001) and was therefore selected as the optimal cut-off for subsequent analyses. AUC, area under the receiver operating characteristic curve; OR, odds ratio.

Given variability in outcomes across stent-size categories, we evaluated candidate cut-offs to identify a clinically interpretable threshold, while accounting for limited sample sizes in extreme categories. The XS category (corresponding to an ICD of ∼20–22 mm) was identified as the optimal threshold, as it demonstrated the highest discriminatory performance for clinical pregnancy. In univariable logistic regression, this threshold was associated with a significantly lower odds of clinical pregnancy (odds ratio [OR] = 0.420, 95% CI 0.262–0.674; *P* < 0.001), and yielded the highest area under the receiver operating characteristic curve (AUC = 0.556), the greatest Youden index (0.113), and the largest likelihood ratio χ^2^ (χ^2^ = 13.044; *P* < 0.001) (Fig. 2; Supplementary Table S5). Patients were subsequently classified as ≤XS or >XS according to stent-size classification. Bootstrap resampling demonstrated the stability of this threshold, with XS being the most frequently selected category (Supplementary Table S6). The median selected threshold was XS (IQR XS–S), with consistent model performance observed in internal validation (Supplementary Table S7).

In addition to evaluating the statistical stability of the selected threshold, we performed a supplementary comparison between the stent-size categories and ultrasound-derived ICD. Because intrauterine stents were placed immediately after adhesiolysis, postoperative ultrasound-derived ICD were used for comparison with the stent-size categories. Agreement was assessed by comparing ultrasound-derived ICD with ICD ranges corresponding to stent size (Supplementary Table S2). Exact agreement was observed in 42.9% of cases, and agreement within ±1 category in 84.1% (weighted κ = 0.539; *P* < 0.001), indicating moderate agreement (Supplementary Table S8).

To explore changes in ultrasound-derived ICD between preoperative and postoperative assessments, we examined women with paired data. The distribution of preoperative and postoperative ultrasound-derived ICD is summarized in Supplementary Table S9. Among the 521 women with paired data, most showed no change in ICD (71.2%), whereas smaller proportions demonstrated increases (12.9%) or decreases (15.9%) (Supplementary Table S10).

### Association between ICD and clinical pregnancy within 1 year of the final hysteroscopy

#### Primary analysis using stent-size classification

Characteristics of patients according to stent-size classification (≤XS vs >XS) are presented in Table 1. Significant differences were observed in menstrual cycle regularity, history of hysteroscopic adhesiolysis, AFS score, DEGO grade, stent placement duration, mode of conception, as well as reproductive outcomes including clinical pregnancy and live birth (Table 1).

In the multivariable logistic regression adjusted for potential confounders, patients in the ≤XS group had significantly lower odds of achieving clinical pregnancy within 1 year of the final hysteroscopy compared with those in the >XS group (adjusted odds ratio [aOR] = 0.447, 95% CI 0.255–0.786; *P* = 0.005). Increasing age (aOR = 0.919, 95% CI 0.877–0.963; *P* < 0.001) and a scarred uterus (aOR = 0.422, 95% CI 0.252–0.705; *P* < 0.001) were independently associated with lower odds of pregnancy. In contrast, a history of one prior missed abortion was associated with higher odds of clinical pregnancy (aOR = 1.885, 95% CI 1.192–2.981; *P* = 0.007). Detailed regression results are presented in Table 2, with the full model shown in Supplementary Table S11 and collinearity diagnostics in Supplementary Table S12.

**Table 2.** Multivariable logistic regression analysis of factors associated with clinical pregnancy within 1 year of the final hysteroscopy.

Variable	aOR	95% CI	*P*
**Baseline characteristics**			
Age (per 1-year increase)	0.919	0.877–0.963	<0.001
Menstrual cycle regularity (irregular vs regular)	1.346	0.774–2.339	0.292
**Past gynecological history**			
Gravidity (reference = 1–2)			
0	0.520	0.128–2.106	0.359
≥3	0.828	0.489–1.403	0.483
Parity (reference = 0)			
1	1.367	0.784–2.381	0.270
≥2	1.184	0.596–2.349	0.630
Induced abortion (reference = 0)			
1	0.720	0.444–1.169	0.184
≥2	0.913	0.514–1.623	0.757
Missed abortion (reference = 0)			
1	1.885	1.192–2.981	0.007
≥2	1.418	0.789–2.545	0.243
Scarred uterus due to prior uterine surgery (Yes vs No)	0.422	0.252–0.705	<0.001
Previous hysteroscopic adhesiolysis (Yes vs No)	0.940	0.622–1.421	0.770
**Treatment-related variables**			
Initial DEGO grade (reference = Grade 1)			
Grade 2	0.864	0.565–1.323	0.502
Grade 3	0.843	0.445–1.599	0.601
Stent-size classification (≤XS vs >XS)	0.447	0.255–0.786	0.005
Second AFS score (per 1-point increase)	0.918	0.782–1.078	0.296
Second DEGO grade (reference = Grade 1)			
Grade 2	0.964	0.618–1.502	0.870
Grade 3	0.573	0.227–1.448	0.239
Mode of conception (ART vs natural conception)	0.793	0.530–1.187	0.259

Variables with *P* < 0.10 in univariable analyses were included in the multivariable logistic regression models. *P* < 0.05 was considered statistically significant. AFS score and grade were defined according to the American Fertility Society classification for intrauterine adhesions (IUA). DEGO grade was defined according to previously published criteria (Zhao *et al*., 2021).

AFS, American Fertility Society; aOR, adjusted odds ratio; DEGO, density of endometrial glandular openings.

#### Supplementary analysis using continuous postoperative ultrasound-derived ICD

Postoperative ultrasound-derived ICD, analyzed as a continuous variable, was positively associated with clinical pregnancy within 1 year of the final hysteroscopy in the multivariable models (aOR = 1.061, 95% CI 1.004–1.120; *P* = 0.035) (Supplementary Table S13), supporting the findings of the primary analysis.

#### Subgroup analyses by conception mode

Subgroup analyses were performed according to mode of conception (natural conception vs ART). The association between stent-size classification (≤XS vs >XS) and clinical pregnancy within 1 year of the final hysteroscopy remained statistically significant in both the natural conception group (aOR = 0.394, 95% CI 0.183–0.846; *P* = 0.017) and the ART group (aOR = 0.451, 95% CI 0.205–0.994; *P* = 0.048). When postoperative ultrasound-derived ICD was analyzed as a continuous variable, a positive association with clinical pregnancy was observed in the natural conception group (aOR = 1.079, 95% CI 1.005–1.160; *P* = 0.037), whereas no statistically significant association was observed in the ART group (aOR = 1.047, 95% CI 0.965–1.135; *P* = 0.273) (Supplementary Tables S14 and S15).

Overall, these findings suggest that the direction of association with clinical pregnancy within 1 year of the final hysteroscopy was consistent across conception modes for both stent-size classification (≤XS vs >XS) and postoperative ultrasound-derived ICD, although statistical significance for the continuous ultrasound-derived ICD was not observed in the ART group.

#### Propensity score-based sensitivity analyses

**Figure 3.**
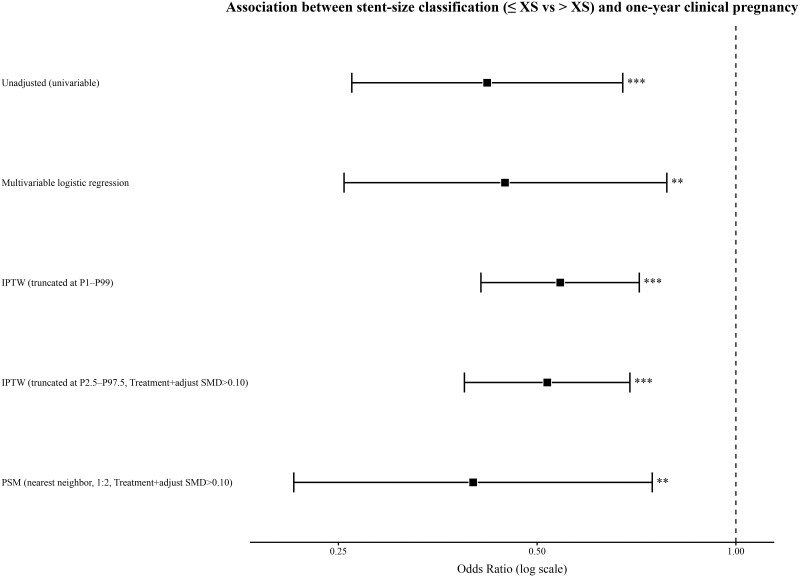
**Association between stent-size classification (≤XS vs >XS) and clinical pregnancy within 1 year of the final hysteroscopy.** Forest plot showing the association between stent-size classification (≤XS vs >XS) and clinical pregnancy within 1 year of the final hysteroscopy. Odds ratios (ORs) with 95% CIs are presented on a logarithmic scale. Estimates were derived from univariable logistic regression, multivariable logistic regression, IPTW with truncation at the 1st–99th percentiles (P1–P99), IPTW with truncation at the 2.5th–97.5th percentiles (P2.5–P97.5) with additional multivariable adjustment for covariates with residual imbalance (SMDs >0.10), and PSM (1:2 nearest-neighbor matching without replacement). The dashed vertical line indicates OR = 1. IPTW, inverse probability of treatment weighting; OR, odds ratio; PSM, propensity score matching; SMD, standardized mean difference. *P < 0.05; **P < 0.01; ***P < 0.001.

To further address potential confounding, we applied IPTW with weight truncation at the P1–P99 and P2.5–P97.5 percentiles, as well as 1:2 nearest-neighbor PSM. After weighting or matching, baseline demographic and gynecological history characteristics were generally well balanced between the ≤XS and >XS groups, with most SMDs < 0.10 (Supplementary Table S16 and Supplementary Fig. S4). Across all analytical approaches, patients in the ≤XS group had consistently lower odds of achieving clinical pregnancy within 1 year of the final hysteroscopy compared with those in the >XS group (IPTW with P1–P99 truncation: aOR = 0.542, 95% CI 0.411–0.714; *P* < 0.001; IPTW with P2.5–P97.5 truncation plus covariate adjustment: aOR = 0.518, 95% CI 0.388–0.691; *P* < 0.001; PSM with covariate adjustment: aOR = 0.400, 95% CI 0.214–0.747; *P* = 0.004) (Table 3 and Fig. 3). Collectively, these findings support the robustness of the association between stent-size classification (≤XS vs >XS) and clinical pregnancy within 1 year of the final hysteroscopy.

**Table 3.** Sensitivity analyses using IPTW and PSM for the association between stent-size classification (≤XS vs >XS) and clinical pregnancy within 1 year of the final hysteroscopy.

Method	aOR	95% CI	*P*
IPTW (P1–P99 truncation)	0.542	0.411–0.714	<0.001
IPTW (P2.5–P97.5 truncation) with covariate adjustment	0.518	0.388–0.691	<0.001
PSM (1:2) with covariate adjustment	0.400	0.214–0.747	0.004

IPTW models used stabilized inverse probability weights with truncation at the 1st–99th (P1–P99) and 2.5th–97.5th (P2.5–P97.5) percentiles. For the PSM analysis, 1:2 nearest-neighbor matching without replacement was applied. Covariate balance was assessed using SMDs, and additional multivariable adjustment was performed when residual imbalance was present (SMD >0.10).

aOR, adjusted odds ratio; IPTW, inverse probability of treatment weighting; PSM, propensity score matchings; SMD, standardized mean difference.

### Association between ICD and secondary reproductive outcomes

#### Live birth following the first clinical pregnancy within 1 year of the final hysteroscopy

Women in the ≤XS group had significantly lower odds of achieving live birth following the first clinical pregnancy within 1 year of the final hysteroscopy compared with those in the >XS group (aOR = 0.500, 95% CI 0.280–0.893; *P* = 0.019). Increasing age and a scarred uterus were independently associated with lower odds of live birth, whereas a history of prior missed abortion was associated with higher odds. When postoperative ultrasound-derived ICD was analyzed as a continuous variable, the direction of association was similar, but it did not reach statistical significance after adjustment (aOR = 1.047, 95% CI 0.993–1.105; *P* = 0.091) (Supplementary Table S17). Given that postoperative fertility behaviors and obstetric management factors were not fully captured, these findings should be interpreted as exploratory.

#### Cumulative live birth within 2 years of the final hysteroscopy

The association between stent-size classification (≤XS vs >XS) and cumulative live birth within 2 years of the final hysteroscopy was directionally consistent but did not reach statistical significance (aOR = 0.606, 95% CI 0.339–1.083; *P* = 0.091). When postoperative ultrasound-derived ICD was analyzed as a continuous variable, no statistically significant association with cumulative live birth was observed in adjusted analyses (aOR = 1.026, 95% CI 0.970–1.084; *P* = 0.371). Although the direction of effect was generally consistent with the findings for clinical pregnancy within 1 year of the final hysteroscopy, neither approach reached statistical significance for cumulative live birth; these results should therefore be interpreted with caution (Supplementary Table S18).

### Time-to-pregnancy analyses

Beyond the binary occurrence of pregnancy, time to conception provides additional insight into reproductive potential. Time-to-pregnancy analyses were therefore performed in 608 women. Kaplan–Meier analysis demonstrated a significantly lower cumulative probability of clinical pregnancy within 1 year of the final hysteroscopy in the ≤XS group compared with the >XS group (log-rank *P* = 0.002; Supplementary Fig. S5). In multivariable Cox proportional hazards analysis, patients in the ≤XS group had a lower hazard of achieving clinical pregnancy compared with those in the >XS group (adjusted hazard ratio [aHR] = 0.630, 95% CI 0.439–0.903; *P* = 0.012). Increasing age (aHR = 0.960 per year, 95% CI 0.935–0.986; *P* = 0.003) and a scarred uterus (aHR = 0.620, 95% CI 0.451–0.853; *P* = 0.003) were independently associated with a lower hazard of clinical pregnancy. In contrast, a history of prior missed abortion was associated with a higher hazard of achieving clinical pregnancy (aHR = 1.332, 95% CI 1.032–1.719; *P* = 0.028) (Supplementary Table S19). The proportional hazards assumption was evaluated using Schoenfeld residuals. The primary exposure (stent-size classification) did not violate the proportional hazards assumption (*P* = 0.26). However, the global test indicated potential deviations from the proportional hazards assumption (*P* = 0.01); therefore, the Cox model results should be interpreted with caution (Supplementary Table S20).

### Incremental predictive value of ICD

To evaluate whether stent-size classification (≤XS vs >XS) and postoperative ultrasound-derived ICD provide prognostic information beyond established clinical variables, we assessed the incremental value of adding these variables to conventional multivariable models for clinical pregnancy within 1 year of the final hysteroscopy.

#### Model performance

Adding stent-size classification (≤XS vs >XS) or postoperative ultrasound-derived ICD separately to the baseline model (including demographic, reproductive, and surgical factors) resulted in only marginal improvements in discrimination. Incorporation of postoperative ultrasound-derived ICD as a continuous variable increased the AUC by 0.008, whereas inclusion of stent-size classification increased the AUC by 0.010; however, neither improvement reached statistical significance (DeLong test, *P* > 0.05) (Supplementary Table S21 and Supplementary Fig. S6). Model calibration was evaluated using bootstrap internal validation (B = 300). All models demonstrated acceptable calibration, with bootstrap-corrected calibration slopes ranging from 0.757 to 0.773 and Brier scores ranging from 0.202 to 0.206 (Supplementary Table S22). Calibration plots showed good agreement between predicted and observed probabilities after bias correction (Supplementary Fig. S7).

#### Clinical utility

**Figure 4.**
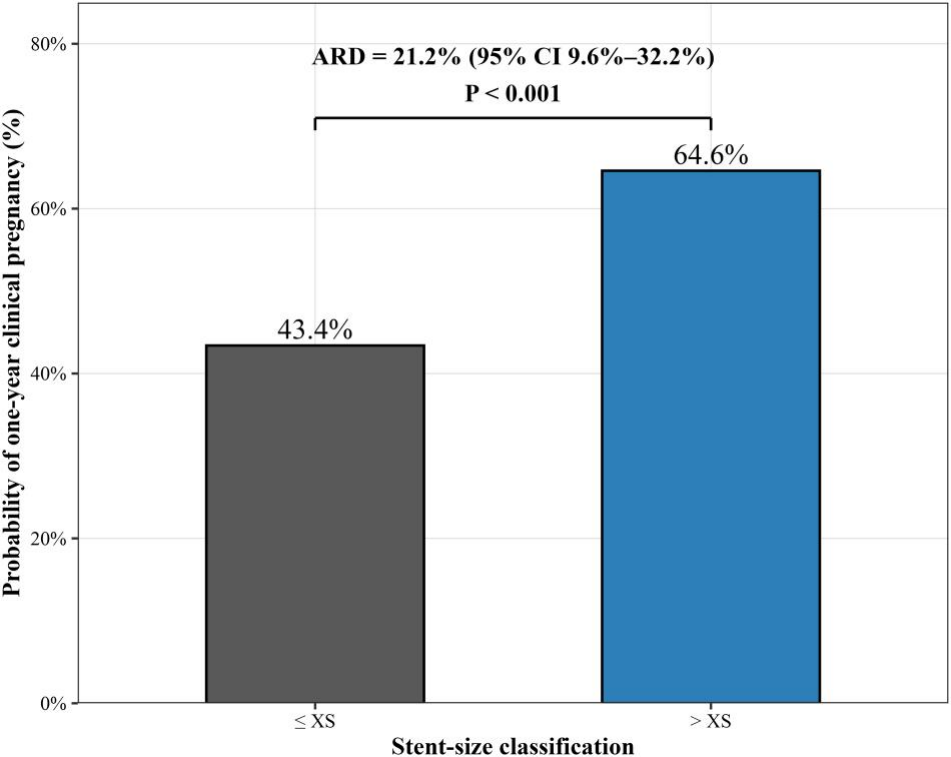
**Absolute difference in the probability of clinical pregnancy within 1 year of the final hysteroscopy between the ≤XS and >XS groups.** The probability of clinical pregnancy within 1 year of the final hysteroscopy was higher in the >XS group than in the ≤XS group (64.6% vs 43.4%). The absolute risk difference (ARD) was 21.2% (95% CI 9.6–32.2; *P* < 0.001). ARD, absolute risk difference.

Regarding clinical utility, the difference in the probability of clinical pregnancy within 1 year of the final hysteroscopy between the ≤XS and >XS groups was 21.2% (ARD = 21.2%, 95% CI 9.6–32.2; *P* < 0.001), corresponding to a NNB of 4.7 (Fig. 4). DCA with bootstrap optimism correction (B = 300) showed comparable net benefit across the three models over a clinically relevant range of threshold probabilities, with no clear separation between the models incorporating postoperative ultrasound-derived ICD or stent-size classification and the baseline model (Supplementary Fig. S8).

Taken together, these findings suggest that, although improvements in model discrimination were modest and overall net benefit remained comparable, stent-size classification (≤XS vs >XS) identifies a clinically meaningful difference in absolute probability of obtaining a clinical pregnancy within 1 year of their final hysteroscopy, supporting its role as a risk stratification marker rather than a standalone predictor.

### Machine learning exploration

To further explore the prognostic contribution of stent-size classification (≤XS vs >XS), a random forest model was developed as an exploratory analysis. The model for predicting clinical pregnancy within 1 year of the final hysteroscopy achieved an AUC of 0.730 in the test set, with an accuracy of 0.701 (95% CI 0.625–0.769), sensitivity of 0.757, specificity of 0.609, and Cohen’s κ of 0.367 (Supplementary Fig. S9A). Variable importance analysis indicated that age was the most influential predictor, with stent-size classification ranking second among the variables included in the model (Supplementary Fig. S9B).

## Discussion

In this retrospective cohort of women with moderate-to-severe IUA undergoing hysteroscopic adhesiolysis with intrauterine stent placement followed by scheduled second-look hysteroscopy, a smaller ICD as reflected by stent-size classification (≤XS vs >XS) was independently associated with a lower probability of clinical pregnancy within 1 year of the final hysteroscopy. These associations remained consistent after multivariable adjustment and propensity score-based analyses and were directionally consistent when analyzed using postoperative ultrasound-derived ICD. Although adding stent-size classification or postoperative ultrasound-derived ICD to conventional models resulted in only marginal improvements in discrimination, a substantial absolute difference in the probability of clinical pregnancy within 1 year of the final hysteroscopy was observed between the ≤XS and >XS groups. Taken together, these findings suggest that stent-size classification (≤XS vs >XS) may serve as a practical risk stratification marker within moderate-to-severe IUA, rather than as a strong standalone predictor of individual-level outcomes.

Reproductive outcomes are generally favorable in women with mild IUA, whereas substantial heterogeneity persists within moderate-to-severe disease despite surgical management, and the AFS classification provides limited discrimination within this spectrum (Cao *et al.*, 2021). Women with comparable baseline severity may therefore experience markedly different reproductive outcomes after similar treatment. In the context of emerging regenerative therapies that are not universally applicable, improved postoperative risk stratification is increasingly important. Our findings suggest that stent-size classification (≤XS vs >XS) may serve as a clinically relevant marker for risk stratification within moderate-to-severe IUA.

A methodological feature of this study is the use of intraoperatively selected stent size as a practical approximation of ICD under direct hysteroscopic visualization. Although ultrasound is the conventional modality for assessing ICD, intraoperative stent selection reflects ICD assessed directly during surgery. The moderate agreement observed between stent-size categories and postoperative ultrasound-derived ICD suggests reasonable concordance between these complementary approaches. Furthermore, the similar direction of association observed when postoperative ultrasound-derived ICD was analyzed as a continuous variable indicates that the overall findings are unlikely to depend on the specific measurement approach.

The improvement in model discrimination after adding stent-size classification (≤XS vs >XS) or postoperative ultrasound-derived ICD to conventional prediction models was limited. Given that reproductive outcomes are influenced by a complex interplay of biological and treatment-related factors, a single parameter is unlikely to substantially enhance overall predictive accuracy. Importantly, discrimination metrics such as the AUC do not fully capture the clinical utility of a prognostic factor. In this context, the observed ARD of 21.2% in the probability of clinical pregnancy within 1 year of the final hysteroscopy between the ≤XS and >XS groups indicates meaningful separation of outcome probabilities. Taken together, these findings suggest that the primary value of stent-size classification may lie in risk stratification rather than in substantially improving individual-level predictive performance.

In subgroup analyses stratified by conception mode, the association between stent-size classification (≤XS vs >XS) and clinical pregnancy within 1 year of the final hysteroscopy remained significant in both the natural conception and ART subgroups, with similar effect directions. By contrast, when postoperative ultrasound-derived ICD was analyzed as a continuous variable, the association was significant only in the natural conception subgroup, whereas the ART subgroup showed a directionally similar but non-significant trend. These findings should be interpreted cautiously, given the potential for confounding arising from differences in reproductive pathway and underlying male factor infertility. Postoperative reproductive planning in our cohort was based on a comprehensive evaluation of both partners. Couples with identifiable infertility factors, including male factor infertility (e.g. abnormal semen parameters), were generally advised to proceed to ART, whereas those without overt infertility attempted natural conception. Consequently, the natural conception subgroup likely included fewer non-uterine fertility barriers, potentially allowing associations involving postoperative ultrasound-derived ICD to be more readily detected. However, detailed quantification of male factor severity and ART-specific variables was unavailable. Outcomes in the ART subgroup may therefore have been influenced by unmeasured factors, including embryo quality and laboratory conditions. Thus, although pathway-based stratification may partially mitigate confounding by indication, residual confounding cannot be excluded. Further studies incorporating detailed male and ART-related data are warranted.

Focusing on live-birth outcomes, we observed that while stent-size classification (≤XS vs >XS) was associated with live birth following the first clinical pregnancy within 1 year of the final hysteroscopy, its association with cumulative live birth within 2 years was weaker and did not reach statistical significance. By contrast, when postoperative ultrasound-derived ICD was analyzed as a continuous variable, no statistically significant associations were observed for either live birth following the first clinical pregnancy or cumulative live birth. This pattern is clinically plausible, as both live-birth outcomes reflect downstream processes beyond the occurrence of clinical pregnancy. Even live birth following the first clinical pregnancy may be influenced by postoperative fertility management, obstetric events, and pregnancy loss, whereas cumulative live birth is further shaped by subsequent reproductive treatment decisions, repeated embryo transfers, and other time-dependent factors. These factors were not comprehensively captured in this retrospective framework and may dilute the observed associations with stent-size classification and postoperative ultrasound-derived ICD. Therefore, the role of stent-size classification and postoperative ultrasound-derived ICD in live birth outcomes, particularly cumulative live birth, should be interpreted cautiously.

From a biological perspective, fibrosis resulting from injury to the endometrial basalis layer is central to the pathogenesis of IUA (Zhao and Hu, 2024) and, in moderate-to-severe cases, may extend to the superficial myometrium. In a separate histopathological study from our center involving an independent cohort of women with moderate-to-severe IUA, myometrial involvement was also observed (Li *et al.*, 2022). These alterations may be associated with changes in uterine cavity architecture and dimensions. However, the present study was not designed to assess histological or mechanistic correlates. Therefore, whether ICD reflects deeper fibrotic involvement, overall disease burden, or broader structural remodeling cannot be determined from the current data and warrants further prospective investigation.

An additional clinical question is whether ICD represents a modifiable therapeutic target. Descriptive comparisons of available pre- and postoperative ultrasound-derived ICD suggested that ICD remained largely stable in most patients, with only a minority showing directional change. However, the present study was not designed to formally evaluate whether changes in ICD are associated with reproductive outcomes. Preoperative ultrasound data were unavailable for some patients because examinations had been performed at outside institutions, and the timing of ultrasound assessments varied according to routine clinical practice rather than a predefined research protocol. Accordingly, ICD in this study should be interpreted primarily as a marker for risk stratification rather than as evidence supporting a modifiable “treat-to-width” therapeutic target.

These findings should be interpreted in light of several limitations. First, the single-center retrospective design may limit generalizability and restrict the availability of certain clinically relevant covariates. Selection bias cannot be excluded, as only women managed under a standardized hysteroscopic protocol were included; patients who did not undergo stent placement, did not complete the planned second-look hysteroscopy, or required additional hysteroscopic procedures beyond the planned two interventions were not included. Although loss to follow-up was moderate (53/613) and sensitivity analyses were performed, attrition bias remains possible. Residual confounding may persist despite multivariable adjustment and pathway-stratified analyses, particularly given the limited availability of detailed ART-related variables, quantitative assessment of male factor infertility, embryo characteristics, and adjunctive treatments.

Measurement-related limitations should also be acknowledged. The nine ordered stent-size categories represent an ordinal rather than a continuous variable, which may introduce boundary misclassification and reduce precision. In addition, the small number of patients in the extreme categories limited the use of the full nine-level ordinal variable in subsequent analyses; accordingly, stent-size classification was dichotomized (≤XS vs >XS). Because this threshold was derived in a data-driven manner, it may be subject to overfitting and optimism. Ultrasound-derived ICD data were retrospectively extracted from the institutional electronic medical record system and recorded as integers after rounding to the nearest millimeter, which may reduce precision. Although a standardized acquisition protocol was used, the timing of ultrasound assessments was not standardized across the menstrual cycle, which may introduce variability in the measurements. Inter-operator variability could not be assessed in this retrospective dataset. Surgical completeness was not formally quantified, and cervical involvement was not systematically recorded; therefore, it could not be evaluated. Finally, although subgroup analyses by conception mode were performed, the available sample size limited the ability to rigorously assess heterogeneity across more specific clinical phenotypes. Prospective multicenter studies with larger cohorts and standardized assessment protocols are warranted, and external validation in independent cohorts is essential before clinical implementation.

From a clinical perspective, stent-size classification (≤XS vs >XS) may help identify women with moderate-to-severe IUA who differ in postoperative reproductive risk. This classification should not be used as a direct decision-making tool but may assist in postoperative risk stratification. Patients in the ≤XS group may warrant closer clinical attention, including more intensive follow-up and timely fertility evaluation if conception does not occur within an expected timeframe. These observations should be interpreted as hypothesis-generating, and external validation in independent cohorts is required before clinical application.

In summary, ICD as reflected by stent-size classification, was associated with clinical pregnancy within 1 year of the final hysteroscopy in women with moderate-to-severe IUA, with directionally consistent findings when analyzed using postoperative ultrasound-derived ICD. Although its contribution to predictive discrimination was limited, it may help distinguish postoperative reproductive risk within this population. The derived classification should be interpreted as cohort-specific rather than a fixed or universal threshold. Further prospective multicenter studies are needed to validate these findings and confirm their clinical relevance.

## Supplementary Material

hoag038_Supplementary_Data

## Data Availability

The patient-level clinical data used in this study are not publicly available due to institutional data security and privacy regulations but may be available from the corresponding author on reasonable request and with permission from the relevant institutional authorities.
