# Supplementary material for: Intercornual distance and postoperative reproductive outcomes in moderate-to-severe intrauterine adhesions: a retrospective cohort study
Source: Hum Reprod Open. 2026 May 5;2026(3):hoag038. doi: 10.1093/hropen/hoag038 (PMC13226053; doi:10.1093/hropen/hoag038)
Supplement: hoag038_Supplementary_Data [file hoag038_supplementary_data.docx]

**Supplementary Materials**

**Supplementary Tables**

**Supplementary Table S1. Distribution of initial AFS scores in the primary analysis cohort.**

| **AFS score** | **n** | **%** |
| --- | --- | --- |
| 5 | 7 | 1.3 |
| 6 | 16 | 2.9 |
| 7 | 22 | 3.9 |
| 8 | 272 | 48.6 |
| 10 | 233 | 41.6 |
| 12 | 10 | 1.8 |
| Total | 560 | 100.0 |

Abbreviations: AFS, American Fertility Society classification system for intrauterine adhesions.

**Supplementary Table S2. Intrauterine stent sizes and corresponding intercornual distance (ICD) ranges.**

| **Stent size** | **Corresponding ICD range (mm)** |
| --- | --- |
| XXXS | 16–17 |
| XXS | 18–19 |
| XS | 20–22 |
| S | 23–25 |
| M | 26–27 |
| L | 28–29 |
| XL | 30–31 |
| XXL | 32–33 |
| XXXL | 34–35 |

Intrauterine stents were manufactured in nine standardized sizes (XXXS–XXXL; see Supplementary Figure S1). These stent sizes were defined based on predefined physical dimensions, and each size corresponds to an approximate intercornual distance (ICD) range derived from the transverse span of the stent when fully expanded within the uterine cavity under hysteroscopic visualization. Stent size selection was primarily guided by preoperative ultrasound-derived ICD, with final determination based on intraoperative hysteroscopic assessment to achieve optimal placement (see Supplementary Figure S2).

**Supplementary Table S3. Baseline characteristics, treatment variables, and reproductive outcomes according to stent-size categories.**

| **Variable** | **XXXS**  **(n = 8)** | **XXS**  **(n = 21)** | **XS**  **(n = 54)** | **S**  **(n = 104)** | **M**  **(n = 167)** | **L**  **(n = 126)** | **XL**  **(n = 51)** | **XXL**  **(n = 20)** | **XXXL**  **(n = 9)** | **P** |
| --- | --- | --- | --- | --- | --- | --- | --- | --- | --- | --- |
| **Baseline characteristics** |  |  |  |  |  |  |  |  |  |  |
| Age, years | 37.6 ± 3.4 | 34.5 ± 5.9 | 34.5 ± 5.4 | 34.2 ± 4.9 | 33.4 ± 4.9 | 33.0 ± 5.2 | 34.0 ± 4.8 | 31.5 ± 3.8 | 33.1 ± 4.2 | 0.049 |
| BMI, kg/m² | 23.6 ± 2.7 | 21.2 ± 3.0 | 22.1 ± 2.7 | 21.6 ± 2.5 | 21.5 ± 2.6 | 22.1 ± 2.8 | 21.9 ± 2.9 | 21.2 ± 3.0 | 21.5 ± 2.0 | 0.246 |
| Ethnicity, n (%) |  |  |  |  |  |  |  |  |  | 0.555 |
| Han Chinese | 6 (75.0%) | 20 (95.2%) | 49 (90.7%) | 94 (90.4%) | 148 (88.6%) | 110 (87.3%) | 48 (94.1%) | 19 (95.0%) | 7 (77.8%) |  |
| Ethnic minorities | 2 (25.0%) | 1 (4.8%) | 5 (9.3%) | 10 (9.6%) | 19 (11.4%) | 16 (12.7%) | 3 (5.9%) | 1 (5.0%) | 2 (22.2%) |  |
| Menstrual cycle regularity, n (%) |  |  |  |  |  |  |  |  |  | 0.391 |
| Regular | 6 (75.0%) | 15 (71.4%) | 42 (77.8%) | 91 (87.5%) | 138 (82.6%) | 111 (88.1%) | 43 (84.3%) | 18 (90.0%) | 8 (88.9%) |  |
| Irregular | 2 (25.0%) | 6 (28.6%) | 12 (22.2%) | 13 (12.5%) | 29 (17.4%) | 15 (11.9%) | 8 (15.7%) | 2 (10.0%) | 1 (11.1%) |  |
| Menstrual duration, n (%) |  |  |  |  |  |  |  |  |  | 0.136 |
| Regular | 8 (100.0%) | 21 (100.0%) | 51 (94.4%) | 99 (95.2%) | 160 (95.8%) | 115 (91.3%) | 45 (88.2%) | 16 (80.0%) | 9 (100.0%) |  |
| Irregular | 0 (0) | 0 (0) | 3 (5.6%) | 5 (4.8%) | 7 (4.2%) | 11 (8.7%) | 6 (11.8%) | 4 (20.0%) | 0 (0) |  |
| **Past gynecological history** |  |  |  |  |  |  |  |  |  |  |
| Gravidity, n (%) |  |  |  |  |  |  |  |  |  | 0.617 |
| 0 | 1 (12.5%) | 0 (0) | 1 (1.9%) | 3 (2.9%) | 3 (1.8%) | 2 (1.6%) | 0 (0) | 0 (0) | 0 (0) |  |
| 1–2 | 4 (50.0%) | 9 (42.9%) | 20 (37.0%) | 46 (44.2%) | 69 (41.3%) | 47 (37.3%) | 17 (33.3%) | 9 (45.0%) | 1 (11.1%) |  |
| ≥ 3 | 3 (37.5%) | 12 (57.1%) | 33 (61.1%) | 55 (52.9%) | 95 (56.9%) | 77 (61.1%) | 34 (66.7%) | 11 (55.0%) | 8 (88.9%) |  |
| Parity, n (%) |  |  |  |  |  |  |  |  |  | 0.002 |
| 0 | 4 (50.0%) | 12 (57.1%) | 32 (59.3%) | 63 (60.6%) | 89 (53.3%) | 53 (42.1%) | 21 (41.2%) | 5 (25.0%) | 1 (11.1%) |  |
| 1 | 4 (50.0%) | 8 (38.1%) | 17 (31.5%) | 29 (27.9%) | 56 (33.5%) | 51 (40.5%) | 18 (35.3%) | 9 (45.0%) | 2 (22.2%) |  |
| ≥ 2 | 0 (0) | 1 (4.8%) | 5 (9.3%) | 12 (11.5%) | 22 (13.2%) | 22 (17.5%) | 12 (23.5%) | 6 (30.0%) | 6 (66.7%) |  |
| Abortion, n (%) |  |  |  |  |  |  |  |  |  | 0.476 |
| 0 | 1 (12.5%) | 0 (0) | 4 (7.4%) | 6 (5.8%) | 6 (3.6%) | 4 (3.2%) | 1 (2.0%) | 0 (0) | 1 (11.1%) |  |
| 1–2 | 5 (62.5%) | 11 (52.4%) | 28 (51.9%) | 62 (59.6%) | 111 (66.5%) | 78 (61.9%) | 30 (58.8%) | 16 (80.0%) | 6 (66.7%) |  |
| ≥ 3 | 2 (25.0%) | 10 (47.6%) | 22 (40.7%) | 36 (34.6%) | 50 (29.9%) | 44 (34.9%) | 20 (39.2%) | 4 (20.0%) | 2 (22.2%) |  |
| Induced abortion, n (%) |  |  |  |  |  |  |  |  |  | 0.773 |
| 0 | 3 (37.5%) | 6 (28.6%) | 24 (44.4%) | 40 (38.5%) | 66 (39.5%) | 48 (38.1%) | 22 (43.1%) | 9 (45.0%) | 3 (33.3%) |  |
| 1 | 2 (25.0%) | 7 (33.3%) | 14 (25.9%) | 30 (28.8%) | 57 (34.1%) | 32 (25.4%) | 17 (33.3%) | 7 (35.0%) | 5 (55.6%) |  |
| ≥ 2 | 3 (37.5%) | 8 (38.1%) | 16 (29.6%) | 34 (32.7%) | 44 (26.3%) | 46 (36.5%) | 12 (23.5%) | 4 (20.0%) | 1 (11.1%) |  |
| Missed abortion, n (%) |  |  |  |  |  |  |  |  |  | 0.385 |
| 0 | 6 (75.0%) | 13 (61.9%) | 20 (37.0%) | 46 (44.2%) | 80 (47.9%) | 63 (50.0%) | 20 (39.2%) | 11 (55.0%) | 4 (44.4%) |  |
| 1 | 1 (12.5%) | 6 (28.6%) | 19 (35.2%) | 36 (34.6%) | 57 (34.1%) | 47 (37.3%) | 15 (29.4%) | 6 (30.0%) | 3 (33.3%) |  |
| ≥ 2 | 1 (12.5%) | 2 (9.5%) | 15 (27.8%) | 22 (21.2%) | 30 (18.0%) | 16 (12.7%) | 16 (31.4%) | 3 (15.0%) | 2 (22.2%) |  |
| Ectopic pregnancy, n (%) |  |  |  |  |  |  |  |  |  | 0.752 |
| No | 6 (75.0%) | 19 (90.5%) | 47 (87.0%) | 93 (89.4%) | 143 (85.6%) | 112 (88.9%) | 44 (86.3%) | 16 (80.0%) | 7 (77.8%) |  |
| Yes | 2 (25.0%) | 2 (9.5%) | 7 (13.0%) | 11 (10.6%) | 24 (14.4%) | 14 (11.1%) | 7 (13.7%) | 4 (20.0%) | 2 (22.2%) |  |
| Biochemical pregnancy, n (%) |  |  |  |  |  |  |  |  |  | 0.998 |
| No | 8 (100.0%) | 19 (90.5%) | 48 (88.9%) | 91 (87.5%) | 147 (88.0%) | 112 (88.9%) | 46 (90.2%) | 18 (90.0%) | 9 (100.0%) |  |
| Yes | 0 (0) | 2 (9.5%) | 6 (11.1%) | 13 (12.5%) | 20 (12.0%) | 14 (11.1%) | 5 (9.8%) | 2 (10.0%) | 0 (0) |  |
| Adenomyosis, n (%) |  |  |  |  |  |  |  |  |  | 0.474 |
| No | 7 (87.5%) | 14 (66.7%) | 36 (66.7%) | 73 (70.2%) | 126 (75.4%) | 97 (77.0%) | 38 (74.5%) | 11 (55.0%) | 7 (77.8%) |  |
| Yes | 1 (12.5%) | 7 (33.3%) | 18 (33.3%) | 31 (29.8%) | 41 (24.6%) | 29 (23.0%) | 13 (25.5%) | 9 (45.0%) | 2 (22.2%) |  |
| Scarred uterus, n (%) |  |  |  |  |  |  |  |  |  | 0.002 |
| No | 6 (75.0%) | 16 (76.2%) | 45 (83.3%) | 81 (77.9%) | 138 (82.6%) | 83 (65.9%) | 35 (68.6%) | 12 (60.0%) | 3 (33.3%) |  |
| Yes | 2 (25.0%) | 5 (23.8%) | 9 (16.7%) | 23 (22.1%) | 29 (17.4%) | 43 (34.1%) | 16 (31.4%) | 8 (40.0%) | 6 (66.7%) |  |
| Previous adhesiolysis, n (%) |  |  |  |  |  |  |  |  |  | < 0.001 |
| No | 1 (12.5%) | 2 (9.5%) | 18 (33.3%) | 54 (51.9%) | 100 (59.9%) | 85 (67.5%) | 37 (72.5%) | 16 (80.0%) | 7 (77.8%) |  |
| Yes | 7 (87.5%) | 19 (90.5%) | 36 (66.7%) | 50 (48.1%) | 67 (40.1%) | 41 (32.5%) | 14 (27.5%) | 4 (20.0%) | 2 (22.2%) |  |
| **Treatment-related variables** |  |  |  |  |  |  |  |  |  |  |
| Initial AFS score | 10 (10–10) | 10 (8–10) | 10 (8–10) | 8 (8–10) | 8 (8–10) | 8 (8–10) | 8 (8–10) | 8 (8–9.5) | 8 (5–8) | 0.002 |
| Initial AFS grade, n (%) |  |  |  |  |  |  |  |  |  | 0.015 |
| Moderate (5–8) | 1 (12.5%) | 9 (42.9%) | 25 (46.3%) | 57 (54.8%) | 103 (61.7%) | 70 (55.6%) | 29 (56.9%) | 15 (75.0%) | 8 (88.9%) |  |
| Severe (9–12) | 7 (87.5%) | 12 (57.1%) | 29 (53.7%) | 47 (45.2%) | 64 (38.3%) | 56 (44.4%) | 22 (43.1%) | 5 (25.0%) | 1 (11.1%) |  |
| Initial DEGO grade, n (%) |  |  |  |  |  |  |  |  |  | < 0.001 |
| Grade 1 | 0 (0) | 1 (4.8%) | 7 (13.0%) | 34 (32.7%) | 72 (43.1%) | 56 (44.4%) | 23 (45.1%) | 11 (55.0%) | 6 (66.7%) |  |
| Grade 2 | 1 (12.5%) | 8 (38.1%) | 29 (53.7%) | 47 (45.2%) | 69 (41.3%) | 63 (50.0%) | 26 (51.0%) | 8 (40.0%) | 2 (22.2%) |  |
| Grade 3 | 7 (87.5%) | 12 (57.1%) | 18 (33.3%) | 23 (22.1%) | 26 (15.6%) | 7 (5.6%) | 2 (3.9%) | 1 (5.0%) | 1 (11.1%) |  |
| Stent placement duration, days | 76  (65–89.25) | 70  (62–87.5) | 70.5  (57–94) | 64  (56–80.75) | 62  (55–79) | 62  (56–73.25) | 57  (54–70) | 60.5  (52.25–77.25) | 51  (47.5–61.5) | < 0.001 |
| Second AFS score | 5 (3.5–5) | 3 (2–3) | 3 (2–3) | 2 (2–3) | 3 (2–3) | 2.5 (2–3) | 2 (2–3) | 2 (0–3) | 2 (0–3) | < 0.001 |
| Second AFS grade, n (%) |  |  |  |  |  |  |  |  |  | < 0.001 |
| 0 | 0 (0) | 4 (19.0%) | 3 (5.6%) | 23 (22.1%) | 24 (14.4%) | 20 (15.9%) | 12 (23.5%) | 6 (30.0%) | 4 (44.4%) |  |
| Mild (1–4) | 2 (25.0%) | 13 (61.9%) | 48 (88.9%) | 75 (72.1%) | 141 (84.4%) | 106 (84.1%) | 39 (76.5%) | 14 (70.0%) | 5 (55.6%) |  |
| Moderate (5–8) | 6 (75.0%) | 4 (19.0%) | 3 (5.6%) | 6 (5.8%) | 2 (1.2%) | 0 (0) | 0 (0) | 0 (0) | 0 (0) |  |
| Second DEGO grade, n (%) |  |  |  |  |  |  |  |  |  | < 0.001 |
| Grade 1 | 0 (0) | 4 (19.0%) | 25 (46.3%) | 64 (61.5%) | 115 (68.9%) | 98 (77.8%) | 39 (76.5%) | 16 (80.0%) | 6 (66.7%) |  |
| Grade 2 | 1 (12.5%) | 6 (28.6%) | 27 (50.0%) | 34 (32.7%) | 49 (29.3%) | 24 (19.0%) | 12 (23.5%) | 4 (20.0%) | 3 (33.3%) |  |
| Grade 3 | 7 (87.5%) | 11 (52.4%) | 2 (3.7%) | 6 (5.8%) | 3 (1.8%) | 4 (3.2%) | 0 (0) | 0 (0) | 0 (0) |  |
| Mode of conception, n (%) |  |  |  |  |  |  |  |  |  | 0.013 |
| Natural conception | 5 (62.5%) | 13 (61.9%) | 19 (35.2%) | 65 (62.5%) | 102 (61.1%) | 78 (61.9%) | 22 (43.1%) | 11 (55.0%) | 4 (44.4%) |  |
| ART | 3 (37.5%) | 8 (38.1%) | 35 (64.8%) | 39 (37.5%) | 65 (38.9%) | 48 (38.1%) | 29 (56.9%) | 9 (45.0%) | 5 (55.6%) |  |
| **Reproductive outcomes** |  |  |  |  |  |  |  |  |  |  |
| Clinical pregnancy (1 year), n (%) | 1 (12.5%) | 9 (42.9%) | 26 (48.1%) | 65 (62.5%) | 108 (64.7%) | 84 (66.7%) | 30 (58.8%) | 16 (80.0%) | 5 (55.6%) | 0.008 |
| Live birth (first pregnancy), n (%) | 0 (0) | 7 (33.3%) | 21 (38.9%) | 53 (51.0%) | 93 (55.7%) | 70 (55.6%) | 24 (47.1%) | 10 (50.0%) | 5 (55.6%) | 0.022 |
| Cumulative live birth (2 years), n (%) | 1 (12.5%) | 8 (38.1%) | 26 (48.1%) | 60 (57.7%) | 102 (61.1%) | 75 (59.5%) | 29 (56.9%) | 10 (50.0%) | 5 (55.6%) | 0.111 |

Values are presented as mean ± standard deviation (SD) or median (interquartile range, IQR) for continuous variables, and as number (percentage) for categorical variables. Continuous variables were compared using one-way analysis of variance (ANOVA) or the Kruskal–Wallis test, as appropriate. Categorical variables were compared using Fisher’s exact test with Monte Carlo simulation. AFS score and grade were defined according to the American Fertility Society classification system for intrauterine adhesions, and DEGO grade according to Zhao et al. (2021). Clinical pregnancy (1 year): clinical pregnancy within one year of the final hysteroscopy; Live birth (first pregnancy): live birth following the first clinical pregnancy within one year of the final hysteroscopy; Cumulative live birth (2 years): cumulative live birth within two years of the final hysteroscopy. P values indicate overall comparisons across stent-size categories.

Abbreviations: AFS, American Fertility Society; DEGO, density of endometrial glandular openings.

**Supplementary Table S4. Trend analyses of reproductive outcomes across ordered stent-size categories.**

| **Reproductive Outcome** | **Linear-by-linear association χ²** | **Degrees of freedom** | **P for trend** | **Kendall’s τ-b** |
| --- | --- | --- | --- | --- |
| Clinical pregnancy (1 year) | 9.36 | 1 | 0.002 | 0.102 |
| Live birth (first pregnancy) | 5.83 | 1 | 0.016 | 0.078 |
| Cumulative live birth (2 years) | 3.51 | 1 | 0.061 | 0.060 |

Clinical pregnancy (1 year): clinical pregnancy within one year of the final hysteroscopy; Live birth (first pregnancy): live birth following the first clinical pregnancy within one year of the final hysteroscopy; Cumulative live birth (2 years): cumulative live birth within two years of the final hysteroscopy. P for trend was calculated using the linear-by-linear association test. Effect size is presented as Kendall’s τ-b. All analyses were performed in the primary cohort (N = 560).

**Supplementary Table S5. Diagnostic performance of stent-size thresholds for predicting clinical pregnancy within one year of the final hysteroscopy.**

| **Threshold (≤ stent-size category)** | **≤ threshold group (n/N, %)** | **Clinical pregnancy rate (≤ vs > threshold)** | **OR (95% CI)** | **P (Wald)** | **AUC (95% CI)** | **Sens** | **Spec** | **Youden J** | **LR χ²** | **df** | **P**  **(LR test)** |
| --- | --- | --- | --- | --- | --- | --- | --- | --- | --- | --- | --- |
| ≤ XXXS | 8 (1.4%) | 12.5% / 62.1% | 0.087 (0.011–0.713) | 0.023 | 0.515 (0.465–0.564) | 0.997 | 0.032 | 0.030 | 8.401 | 1 | 0.004 |
| ≤ XXS | 29 (5.2%) | 34.5% / 62.9% | 0.310 (0.141–0.681) | 0.004 | 0.529 (0.480–0.579) | 0.971 | 0.088 | 0.059 | 9.072 | 1 | 0.003 |
| ≤ XS | 83 (14.8%) | 43.4% / 64.6% | 0.420 (0.262–0.674) | < 0.001 | 0.556 (0.507–0.606) | 0.895 | 0.218 | 0.113 | 13.044 | 1 | < 0.001 |
| ≤ S | 187 (33.4%) | 54.0% / 65.1% | 0.628 (0.439–0.899) | 0.011 | 0.552 (0.503–0.602) | 0.706 | 0.398 | 0.105 | 6.464 | 1 | 0.011 |
| ≤ M | 354 (63.2%) | 59.0% / 65.5% | 0.758 (0.530–1.083) | 0.128 | 0.532 (0.483–0.581) | 0.392 | 0.671 | 0.064 | 2.334 | 1 | 0.127 |
| ≤ L | 480 (85.7%) | 61.0% / 63.7% | 0.891 (0.545–1.456) | 0.645 | 0.507 (0.458–0.556) | 0.148 | 0.866 | 0.014 | 0.214 | 1 | 0.644 |
| ≤ XL | 531 (94.8%) | 60.8% / 72.4% | 0.592 (0.257–1.360) | 0.217 | 0.512 (0.463–0.561) | 0.061 | 0.963 | 0.024 | 1.627 | 1 | 0.202 |
| ≤ XXL | 551 (98.4%) | 55.6% / 61.5% | 1.279 (0.340–4.817) | 0.716 | 0.502 (0.453–0.551) | 0.985 | 0.019 | 0.004 | 0.131 | 1 | 0.717 |

Each stent-size threshold defines dichotomization into ≤ and > categories according to stent size. P values were derived from univariable logistic regression comparing clinical pregnancy between groups. AUC, sensitivity, specificity, and Youden J were calculated based on the corresponding binary classification. The P value next to OR corresponds to the Wald test, whereas the P value associated with LR χ² corresponds to the likelihood-ratio test for model fit.

Abbreviations: AUC, area under the receiver operating characteristic curve; CI, confidence interval; df, degrees of freedom; LR χ², likelihood ratio chi-square; OR, odds ratio.

**Supplementary Table S6. Distribution of selected thresholds across bootstrap samples.**

| Threshold (stent-size category) | Frequency (n) | % |
| --- | --- | --- |
| XS | 152 | 50.7 |
| M | 64 | 21.3 |
| XXS | 42 | 14.0 |
| S | 35 | 11.7 |
| XL | 4 | 1.3 |
| L | 2 | 0.7 |
| XXXS | 1 | 0.3 |

Threshold stability was assessed using bootstrap resampling (300 iterations) in the primary analysis cohort. In each resample, all possible dichotomizations of stent-size categories (≤ threshold vs > threshold) were evaluated, and the optimal threshold was defined as the category yielding the highest training AUC within a multivariable logistic regression model including prespecified baseline covariates (age, BMI, scarred uterus, recurrent intrauterine adhesions, initial AFS score, and mode of conception). Frequencies represent the number of bootstrap samples in which each threshold was selected as optimal, and percentages are based on 300 bootstrap iterations.

**Supplementary Table S7. Optimal threshold and internally validated model performance.**

| Measure | Value |
| --- | --- |
| Median selected threshold (IQR) | XS (XS–S) |
| Apparent AUC | 0.766 |
| Mean optimism | 0.043 |
| Optimism-corrected AUC | 0.672 |
| Bootstrap iterations | 300 |

Threshold selection and internal validation were performed using bootstrap resampling (300 iterations). In each resample, candidate thresholds were evaluated within a multivariable logistic regression model including prespecified baseline covariates (age, BMI, scarred uterus, recurrent intrauterine adhesions, initial AFS score, and mode of conception). The optimal threshold was defined as the category yielding the highest training AUC. Apparent AUC represents model performance in the bootstrap samples, and optimism-corrected AUC reflects the internally validated estimate after adjustment for overfitting.

**Supplementary Table S8. Agreement between stent-size categories and postoperative ultrasound-derived intercornual distance (ICD) (N = 560).**

| Measure | Value |
| --- | --- |
| Exact agreement (no category difference) | 240 (42.9%) |
| ± 1-category difference | 231 (41.3%) |
| ≥ 2-category difference | 85 (15.2%) |
| Outside predefined stent-size ranges | 4 (0.7%) |
| Weighted Cohen’s κ | 0.539 |
| P value | < 0.001 |

Values are presented as n (%) or as indicated. Stent-size categories (XXXS–XXXL) represent nine ordered groups defined by standardized stent dimensions, each corresponding to an approximate intercornual distance (ICD) range derived from the transverse span of the stent (Supplementary Table S2). Postoperative ultrasound-derived ICD (mm) was compared with the corresponding stent-size ICD ranges. Exact agreement was defined as an ultrasound value falling within the corresponding stent-size ICD range. A ± 1-category difference was defined as an ultrasound value falling within an adjacent range, whereas a ≥ 2-category difference indicated a larger discrepancy. Values outside all stent-size ICD ranges were classified as outside range. Agreement within ± 1-category (including exact agreement) was 84.1% (471/560). Weighted Cohen’s κ was calculated using quadratic weights.

**Supplementary Table S9. Timing of ultrasound examinations and ultrasound-derived intercornual distance.**

| Ultrasound examination | N | Time from surgery (days) | Range (days) | ICD (mm) | Range (mm) |
| --- | --- | --- | --- | --- | --- |
| Preoperative ultrasound | 521† | 14.62 (0.57–22.59) | 0.27–226.62 | 25 (22–28) | 13–39 |
| Postoperative ultrasound | 560 | 1.39 (1.36–1.48) | 0.36–93.2 | 26 (23–28) | 16–43 |

† Preoperative ultrasound data were available for 521 patients. For the remaining patients, preoperative ultrasound examinations were performed at outside institutions and data were not available for retrospective review. Time from surgery represents the interval between ultrasound examination and the initial hysteroscopic (stent placement) procedure (preoperative: surgery date minus ultrasound date; postoperative: ultrasound date minus surgery date). Values are presented as median (interquartile range, IQR).

**Supplementary Table S10. Distribution of changes in ultrasound-derived intercornual distance (ICD) among patients with paired preoperative and postoperative assessments (n = 521).**

| Change in ICD (mm) | n | % |
| --- | --- | --- |
| No change | 371 | 71.2 |
| Increase | 67 | 12.9 |
| 1 mm | 29 | 5.6 |
| 2 mm | 13 | 2.5 |
| 3 mm | 13 | 2.5 |
| ≥ 4 mm | 12 | 2.3 |
| Decrease | 83 | 15.9 |
| 1 mm | 41 | 7.9 |
| ≥ 2 mm | 42 | 8.1 |

Change in ICD was defined as postoperative minus preoperative values. Positive values indicate an increase, and negative values indicate a decrease. ICD values were recorded as whole millimeters in routine clinical practice and were extracted as such for this retrospective analysis. Values are presented as n (%). Only patients with paired preoperative and postoperative assessments (n = 521) were included.

**Supplementary Table S11. Univariable logistic regression analyses of factors associated with clinical pregnancy within one year of the final hysteroscopy.**

| Variable | OR | 95% CI | P |
| --- | --- | --- | --- |
| Baseline characteristics |  |  |  |
| Age (per 1-year increase) | 0.900 | 0.868–0.933 | < 0.001 |
| BMI (kg/m²) | 1.003 | 0.941–1.069 | 0.924 |
| Ethnicity (Ethnic minorities vs Han Chinese) | 0.775 | 0.450–1.337 | 0.360 |
| Menstrual cycle regularity (Irregular vs Regular) | 1.713 | 1.041–2.820 | 0.034 |
| Menstrual duration (Irregular vs Regular) | 0.772 | 0.391–1.524 | 0.455 |
| Past gynecological history |  |  |  |
| Gravidity (reference = 1–2) |  |  |  |
| 0 | 0.432 | 0.121–1.543 | 0.196 |
| ≥ 3 | 0.552 | 0.385–0.792 | 0.001 |
| Parity (reference = 0) |  |  |  |
| 1 | 0.557 | 0.381–0.814 | 0.003 |
| ≥ 2 | 0.423 | 0.258–0.693 | < 0.001 |
| Abortion (reference = 0) |  |  |  |
| 1–2 | 1.649 | 0.707–3.846 | 0.247 |
| ≥ 3 | 1.234 | 0.518–2.936 | 0.635 |
| Induced abortion (reference = 0) |  |  |  |
| 1 | 0.609 | 0.402–0.923 | 0.020 |
| ≥ 2 | 0.577 | 0.380–0.875 | 0.010 |
| Missed abortion (reference = 0) |  |  |  |
| 1 | 2.060 | 1.386–3.062 | < 0.001 |
| ≥ 2 | 1.351 | 0.854–2.138 | 0.199 |
| Ectopic pregnancy (Yes vs No) | 0.946 | 0.572–1.564 | 0.828 |
| Biochemical pregnancy (Yes vs No) | 0.854 | 0.500–1.460 | 0.564 |
| Adenomyosis (Yes vs No) | 1.133 | 0.770–1.667 | 0.526 |
| Scarred uterus due to prior uterine surgery (Yes vs No) | 0.381 | 0.258–0.563 | < 0.001 |
| Previous hysteroscopic adhesiolysis (Yes vs No) | 0.683 | 0.484–0.963 | 0.030 |
| Treatment-related variables |  |  |  |
| Initial AFS score (per 1-point increase) | 0.965 | 0.844–1.103 | 0.597 |
| Initial DEGO grade (reference = Grade 1) |  |  |  |
| Grade 2 | 0.749 | 0.511–1.098 | 0.138 |
| Grade 3 | 0.521 | 0.318–0.851 | 0.009 |
| Stent-size classification (≤ XS vs > XS) | 0.420 | 0.262–0.674 | < 0.001 |
| Stent placement duration (days) | 0.998 | 0.991–1.005 | 0.548 |
| Second AFS score (per 1-point increase) | 0.880 | 0.769–1.008 | 0.064 |
| Second DEGO grade (reference = Grade 1) |  |  |  |
| Grade 2 | 0.725 | 0.496–1.059 | 0.097 |
| Grade 3 | 0.348 | 0.168–0.723 | 0.005 |
| Mode of conception (ART vs natural conception) | 0.575 | 0.407–0.811 | 0.002 |

Abbreviations: AFS, American Fertility Society; CI, confidence interval; DEGO, density of endometrial glandular openings; OR, odds ratio.

**Supplementary Table S12. Collinearity diagnostics for variables included in the multivariable logistic regression model.**

| Variable | Tolerance | VIF |
| --- | --- | --- |
| Age | 0.649 | 1.540 |
| Menstrual cycle regularity | 0.929 | 1.076 |
| Gravidity | 0.772 | 1.295 |
| Parity | 0.574 | 1.741 |
| Induced abortion | 0.757 | 1.322 |
| Missed abortion | 0.825 | 1.211 |
| Scarred uterus due to prior uterine surgery | 0.736 | 1.359 |
| Previous hysteroscopic adhesiolysis | 0.809 | 1.236 |
| Initial DEGO grade | 0.724 | 1.381 |
| Stent-size classification | 0.796 | 1.256 |
| Second AFS score | 0.850 | 1.176 |
| Second DEGO grade | 0.711 | 1.407 |
| Mode of conception | 0.833 | 1.200 |

Only variables with P < 0.10 in the univariable analyses were entered into the multivariable logistic regression model. Collinearity diagnostics were performed for all variables included in the multivariable model. Tolerance and variance inflation factor (VIF) were calculated, and no evidence of multicollinearity was observed (all VIF < 5).

Abbreviations: AFS, American Fertility Society; DEGO, density of endometrial glandular openings; VIF, variance inflation factor.

**Supplementary Table S13. Multivariable logistic regression analysis of factors associated with clinical pregnancy within one year of the final hysteroscopy using continuous postoperative ultrasound-derived ICD.**

| Variable | aOR | 95% CI | P |
| --- | --- | --- | --- |
| Baseline characteristics |  |  |  |
| Age (per 1-year increase) | 0.917 | 0.875–0.961 | < 0.001 |
| Menstrual cycle regularity  (Irregular vs Regular) | 1.252 | 0.725–2.161 | 0.420 |
| Past gynecological history |  |  |  |
| Gravidity (reference = 1–2) |  |  |  |
| 0 | 0.515 | 0.128–2.072 | 0.350 |
| ≥ 3 | 0.823 | 0.486–1.391 | 0.466 |
| Parity (reference = 0) |  |  |  |
| 1 | 1.351 | 0.778–2.345 | 0.286 |
| ≥ 2 | 1.217 | 0.615–2.406 | 0.573 |
| Induced abortion (reference = 0) |  |  |  |
| 1 | 0.723 | 0.446–1.172 | 0.188 |
| ≥ 2 | 0.923 | 0.520–1.637 | 0.783 |
| Missed abortion (reference = 0) |  |  |  |
| 1 | 1.917 | 1.213–3.030 | 0.005 |
| ≥ 2 | 1.366 | 0.761–2.450 | 0.296 |
| Scarred uterus due to prior uterine surgery (Yes vs No) | 0.424 | 0.253–0.708 | 0.001 |
| Previous hysteroscopic adhesiolysis (Yes vs No) | 0.942 | 0.622–1.425 | 0.776 |
| Treatment-related variables |  |  |  |
| Initial DEGO grade (reference = Grade 1) |  |  |  |
| Grade 2 | 0.851 | 0.556–1.301 | 0.456 |
| Grade 3 | 0.820 | 0.434–1.546 | 0.539 |
| Postoperative ultrasound-derived ICD (per 1-mm increase) | 1.061 | 1.004–1.120 | 0.035 |
| Second AFS score (per 1-point increase) | 0.909 | 0.775–1.066 | 0.239 |
| Second DEGO grade (reference = Grade 1) |  |  |  |
| Grade 2 | 0.947 | 0.610–1.471 | 0.809 |
| Grade 3 | 0.504 | 0.201–1.261 | 0.143 |
| Mode of conception (ART vs natural conception) | 0.765 | 0.512–1.143 | 0.191 |

In this analysis, postoperative ultrasound ICD (per 1-mm increase) was included as a continuous variable instead of the stent-size classification used in the primary model, while other covariates remained the same as in the main multivariable model.

Abbreviations: AFS, American Fertility Society; aOR, adjusted odds ratio; CI, confidence interval; DEGO, density of endometrial glandular openings; ICD, intercornual distance.

**Supplementary Table S14. Multivariable logistic regression analyses of factors associated with clinical pregnancy within one year of the final hysteroscopy in the natural conception group (N = 319).**

| Variable | aOR (95% CI) | P |
| --- | --- | --- |
| Model 1: Baseline + Stent-size classification |  |  |
| Stent-size classification (≤ XS vs > XS) | 0.394 (0.183–0.846) | 0.017 |
| Model 2: Baseline + Postoperative ultrasound ICD |  |  |
| Postoperative ultrasound-derived ICD (per 1-mm increase) | 1.079 (1.005–1.160) | 0.037 |

Models were adjusted for covariates with P < 0.10 in univariable analyses within this subgroup.

Abbreviations: aOR, adjusted odds ratio; CI, confidence interval; ICD, intercornual distance.

**Supplementary Table S15. Multivariable logistic regression analyses of factors associated with clinical pregnancy within one year of the final hysteroscopy in the ART group (N = 241).**

| Variable | aOR (95% CI) | P |
| --- | --- | --- |
| Model 1: Baseline + Stent-size classification |  |  |
| Stent-size classification (≤ XS vs > XS) | 0.451 (0.205–0.994) | 0.048 |
| Model 2: Baseline + Postoperative ultrasound ICD |  |  |
| Postoperative ultrasound-derived ICD (per 1-mm increase) | 1.047 (0.965–1.135) | 0.273 |

Models were adjusted for covariates with P < 0.10 in univariable analyses within this subgroup.

Abbreviations: aOR, adjusted odds ratio; CI, confidence interval; ICD, intercornual distance.

**Supplementary Table S16. Standardized mean differences (SMDs) before and after weighting or matching.**

| **Variable** | **Before weighting** | **IPTW (P1–P99)** | **IPTW (P2.5–P97.5)** | **PSM (1:2)** |
| --- | --- | --- | --- | --- |
| **Demographics** |  |  |  |  |
| Age (years) | 0.092 | 0.031 | 0.047 | 0.038 |
| BMI (kg/m²) | 0.018 | 0.020 | 0.010 | 0.116 |
| Ethnicity (Han / Others) | 0.034 | 0.049 | 0.030 | 0.020 |
| Menstrual cycle regularity (Regular / Irregular) | 0.270 | 0.067 | 0.036 | 0.183 |
| Menstrual duration (Regular / Irregular) | 0.135 | 0.047 | 0.009 | 0.073 |
| **Reproductive history** |  |  |  |  |
| Gravidity (0 / 1–2 / ≥ 3) | 0.020 | 0.080 | 0.015 | 0.017 |
| Parity (0 / 1 / ≥ 2) | 0.398 | 0.069 | 0.078 | 0.012 |
| Abortion (0 / 1–2 / ≥ 3) | 0.080 | 0.079 | 0.077 | 0.086 |
| Induced abortion (0 / 1 / ≥ 2) | 0.029 | 0.026 | 0.027 | 0.041 |
| Missed abortion (0 / 1 / ≥ 2) | 0.031 | 0.038 | 0.038 | 0.087 |
| Ectopic pregnancy (No / Yes) | 0.008 | 0.074 | 0.049 | 0.116 |
| Biochemical pregnancy (No / Yes) | 0.054 | 0.070 | 0.050 | 0.020 |
| **Past gynecological history** |  |  |  |  |
| Adenomyosis (No / Yes) | 0.115 | 0.016 | 0.010 | 0.039 |
| Scarred uterus due to prior uterine surgery (No / Yes) | 0.160 | 0.094 | 0.105 | 0.015 |
| Previous hysteroscopic adhesiolysis (No / Yes) | 0.755 | 0.011 | 0.083 | 0.014 |

Standardized mean differences (SMDs) were used to assess covariate balance between the ≤ XS group and the > XS group before and after adjustment. Inverse probability of treatment weighting (IPTW) was applied using two truncation strategies: the 1st–99th percentile (P1–P99) and the 2.5th–97.5th percentile (P2.5–P97.5). A 1:2 nearest-neighbor propensity score matching (PSM) without replacement was also performed for comparison. An absolute SMD of < 0.10 was considered indicative of adequate covariate balance.

**Supplementary Table S17. Multivariable logistic regression analyses of factors associated with live birth following the first clinical pregnancy within one year of the final hysteroscopy.**

| Variable | aOR (95% CI) | P |
| --- | --- | --- |
| Model 1: Stent-size classification |  |  |
| Age (per 1-year increase) | 0.909 (0.870–0.950) | < 0.001 |
| Scarred uterus due to prior uterine surgery (Yes vs No) | 0.564 (0.334–0.951) | 0.032 |
| Missed abortion (reference = 0) |  |  |
| 1 | 2.016 (1.292–3.144) | 0.002 |
| ≥ 2 | 1.853 (1.038–3.308) | 0.037 |
| Stent-size classification (≤ XS vs > XS) | 0.500 (0.280–0.893) | 0.019 |
| Model 2: Postoperative ultrasound ICD |  |  |
| Age (per 1-year increase) | 0.906 (0.867–0.947) | < 0.001 |
| Scarred uterus due to prior uterine surgery (Yes vs No) | 0.563 (0.334–0.950) | 0.031 |
| Missed abortion (reference = 0) |  |  |
| 1 | 2.052 (1.316–3.198) | 0.002 |
| ≥ 2 | 1.813 (1.016–3.235) | 0.044 |
| Postoperative ultrasound-derived ICD (per 1-mm increase) | 1.047 (0.993–1.105) | 0.091 |

Multivariable logistic regression analysis was performed for live birth following the first clinical pregnancy within one year of the final hysteroscopy. Variables with P < 0.10 in univariable analyses were selected as covariates. Two alternative multivariable models were fitted by including either stent-size classification (≤ XS vs >XS) or postoperative ultrasound-derived ICD (per 1 mm increase), while keeping the same set of selected covariates.

Abbreviations: aOR, adjusted odds ratio; CI, confidence interval; ICD, intercornual distance.

**Supplementary Table S18. Multivariable logistic regression analyses of factors associated with cumulative live birth within two years of the final hysteroscopy.**

| Variable | aOR (95% CI) | P |
| --- | --- | --- |
| Model 1: Stent-size classification |  |  |
| Age (per 1-year increase) | 0.883 (0.842–0.925) | < 0.001 |
| Scarred uterus due to prior uterine surgery (Yes vs No) | 0.581 (0.345–0.976) | 0.040 |
| Missed abortion (reference = 0) |  |  |
| 1 | 1.851 (1.171–2.928) | 0.008 |
| ≥ 2 | 1.974 (1.085–3.592) | 0.026 |
| Stent-size classification (≤ XS vs > XS) | 0.606 (0.339–1.083) | 0.091 |
| Stent placement duration (days) | 0.990 (0.982–0.999) | 0.021 |
| Model 2: Postoperative ultrasound ICD |  |  |
| Age (per 1-year increase) | 0.882 (0.842–0.925) | < 0.001 |
| Scarred uterus due to prior uterine surgery (Yes vs No) | 0.583 (0.347–0.980) | 0.042 |
| Missed abortion (reference = 0) |  |  |
| 1 | 1.869 (1.182–2.954) | 0.007 |
| ≥ 2 | 1.932 (1.063–3.512) | 0.031 |
| Postoperative ultrasound-derived ICD (per 1-mm increase) | 1.026 (0.970–1.084) | 0.371 |
| Stent placement duration (days) | 0.990 (0.982–0.998) | 0.020 |

Multivariable logistic regression analysis was performed for cumulative live birth within two years of the final hysteroscopy. Variables with P < 0.10 in univariable analyses were selected as covariates. Two alternative multivariable models were fitted by including either stent-size classification (≤ XS vs > XS) or postoperative ultrasound-derived ICD (per 1-mm increase), while keeping the same set of selected covariates.

Abbreviations: aOR, adjusted odds ratio; CI, confidence interval; ICD, intercornual distance.

**Supplementary Table S19. Multivariable Cox proportional hazards regression for time to clinical pregnancy within one year** **of the final hysteroscopy.**

| Variable | aHR | 95% CI | P |
| --- | --- | --- | --- |
| Baseline characteristics |  |  |  |
| Age (per 1-year increase) | 0.960 | 0.935–0.986 | 0.003 |
| Menstrual cycle regularity (Irregular vs Regular) | 1.243 | 0.941–1.641 | 0.125 |
| Past gynecological history |  |  |  |
| Gravidity (reference = 1–2) |  |  |  |
| 0 | 1.045 | 0.413–2.644 | 0.926 |
| ≥ 3 | 0.898 | 0.664–1.215 | 0.487 |
| Parity (reference = 0) |  |  |  |
| 1 | 1.244 | 0.914–1.693 | 0.165 |
| ≥ 2 | 1.021 | 0.667–1.564 | 0.922 |
| Induced abortion (reference = 0) |  |  |  |
| 1 | 0.954 | 0.726–1.252 | 0.733 |
| ≥ 2 | 0.908 | 0.650–1.268 | 0.571 |
| Missed abortion (reference = 0) |  |  |  |
| 1 | 1.332 | 1.032–1.719 | 0.028 |
| ≥ 2 | 1.335 | 0.940–1.897 | 0.106 |
| Scarred uterus due to prior uterine surgery (Yes vs No) | 0.620 | 0.451–0.853 | 0.003 |
| Treatment-related variables |  |  |  |
| Initial DEGO grade (reference = Grade 1) |  |  |  |
| Grade 2 | 0.857 | 0.680–1.081 | 0.192 |
| Grade 3 | 0.673 | 0.465–0.972 | 0.035 |
| Stent-size classification (≤ XS vs > XS) | 0.630 | 0.439–0.903 | 0.012 |
| Second DEGO grade (reference = Grade 1) |  |  |  |
| Grade 2 | 1.100 | 0.855–1.417 | 0.458 |
| Grade 3 | 0.731 | 0.389–1.372 | 0.330 |
| Mode of conception (ART vs natural conception) | 0.950 | 0.747–1.208 | 0.677 |

Multivariable Cox proportional hazards regression analysis was performed for time to clinical pregnancy within one year of the final hysteroscopy. Variables with P < 0.10 in univariable analyses were selected as covariates. The proportional hazards assumption was assessed using Schoenfeld residuals. The composite abortion variable was not included in the multivariable model to avoid overlap with induced abortion and missed abortion.

Abbreviations: aHR, adjusted hazard ratio; CI, confidence interval; DEGO, density of endometrial glandular openings.

**Supplementary Table S20. Assessment of the proportional hazards assumption using Schoenfeld residuals in the multivariable Cox proportional hazards model.**

| Variable | Chi-square | df | P |
| --- | --- | --- | --- |
| Age | 0.00 | 1 | 0.995 |
| Menstrual cycle regularity | 1.29 | 1 | 0.255 |
| Gravidity | 3.33 | 2 | 0.189 |
| Parity | 7.31 | 2 | 0.026 |
| Induced abortion | 1.04 | 2 | 0.595 |
| Missed abortion | 0.57 | 2 | 0.754 |
| Scarred uterus | 0.25 | 1 | 0.620 |
| Initial DEGO grade | 9.24 | 2 | 0.010 |
| Stent-size classification (≤ XS vs > XS) | 1.27 | 1 | 0.260 |
| Second DEGO grade | 3.02 | 2 | 0.221 |
| Mode of conception | 9.15 | 1 | 0.002 |
| Global test | 33.22 | 17 | 0.011 |

The proportional hazards assumption was assessed using Schoenfeld residuals. A P < 0.05 suggests potential violation of the proportional hazards assumption.

Abbreviations: DEGO, density of endometrial glandular openings; df, degrees of freedom.

**Supplementary Table S21. Discrimination performance of prediction models for clinical pregnancy within one year of the final hysteroscopy.**

| Model | AUC (95% CI) | ΔAUC | DeLong p |
| --- | --- | --- | --- |
| Baseline | 0.725 (0.682–0.768) | Reference |  |
| Baseline + Postoperative ultrasound-derived ICD | 0.733 (0.690–0.776) | +0.008 | 0.167 |
| Baseline + Stent-size classification (≤ XS vs > XS) | 0.735 (0.692–0.777) | +0.010 | 0.166 |

The baseline model included demographic factors (age, body mass index, ethnicity), medical history (uterine fibroids, adenomyosis, recurrent IUA), reproductive history (gravidity, parity, missed abortion, ectopic pregnancy, biochemical pregnancy), menstrual characteristics, surgical factors (DEGO and AFS grades), and planned conception method. Differences in AUC between models were compared using the DeLong test.

Abbreviations: AUC, area under the receiver operating characteristic curve; CI, confidence interval; ICD, intercornual distance.

**Supplementary Table S22. Calibration performance of predictive models for clinical pregnancy within one year of the final hysteroscopy.**

| Model | Calibration slope (bootstrap-corrected) | Brier score |
| --- | --- | --- |
| Baseline | 0.771 | 0.206 |
| Baseline + Postoperative ultrasound-derived ICD | 0.757 | 0.204 |
| Baseline + Stent-size classification (≤ XS vs > XS) | 0.773 | 0.202 |

Calibration performance was assessed using bootstrap internal validation (B = 300). Calibration slopes closer to 1 indicate better agreement between predicted and observed probabilities. The Brier score reflects overall prediction error, with lower values indicating better model performance.

**Supplementary Figures**


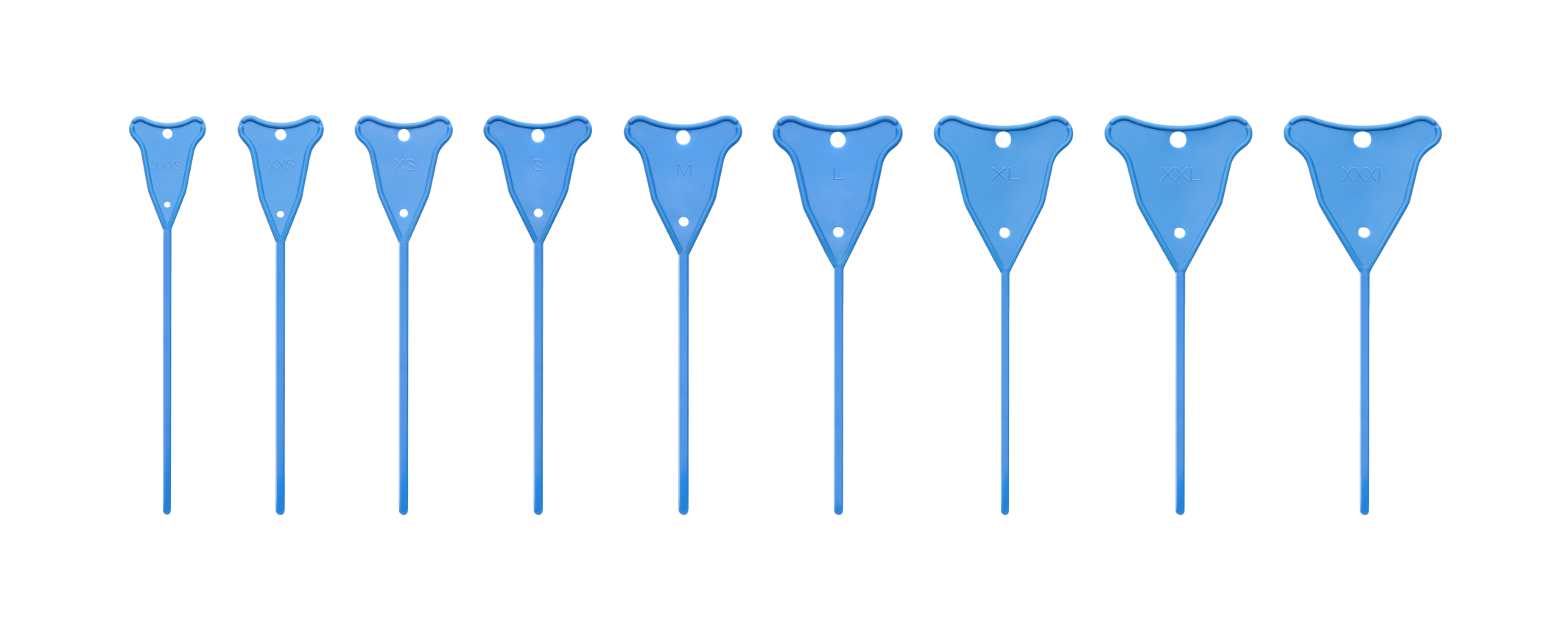


**Supplementary Figure S1. Morphology and size categories of intrauterine stents.**

The figure shows the available intrauterine stent size categories ranging from XXXS to XXXL (from left to right). The stents are made of medical-grade silicone and have a uterine-shaped ring structure with a cylindrical tail to facilitate removal. The central portion is designed as a thin layer to minimize endometrial compression, whereas the peripheral rim is thickened to provide structural support. Two openings allow menstrual outflow. Stent size was selected based on preoperative ultrasound-derived intercornual distance (ICD), with final determination based on intraoperative hysteroscopic assessment.


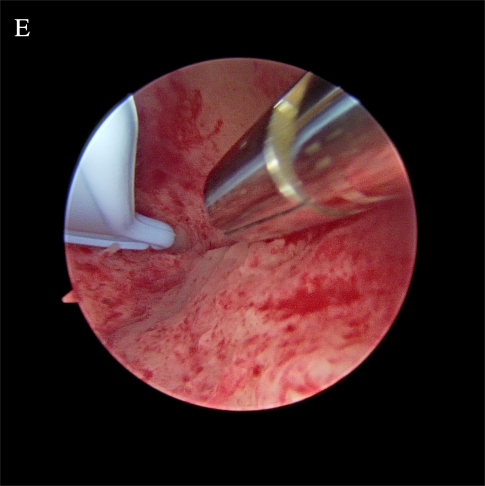

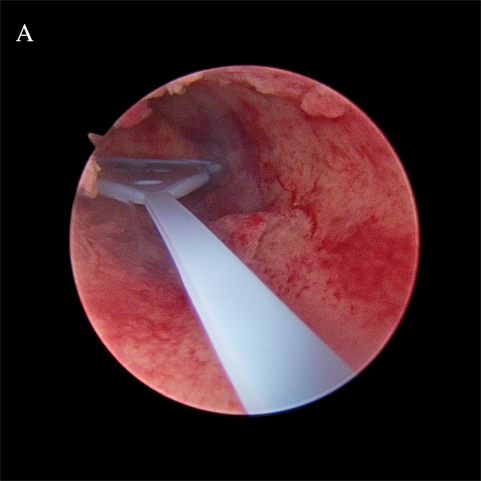

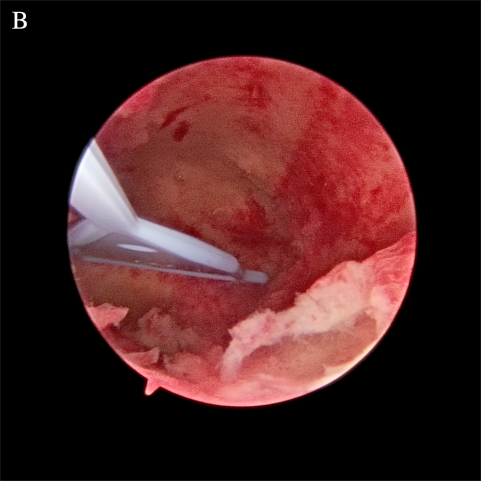

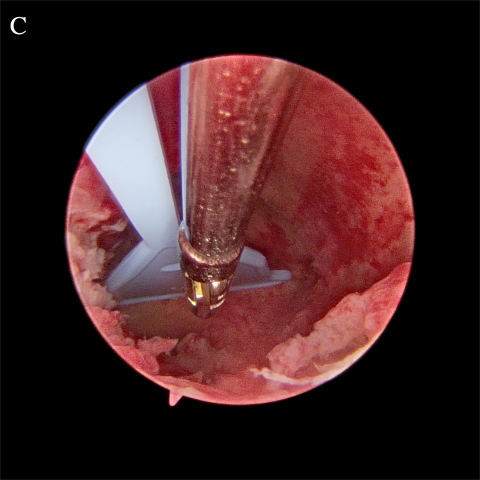

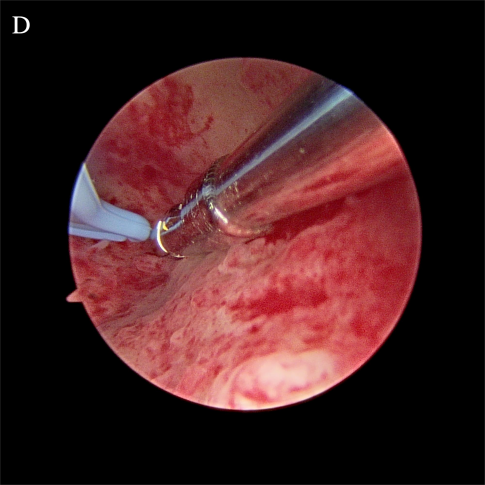

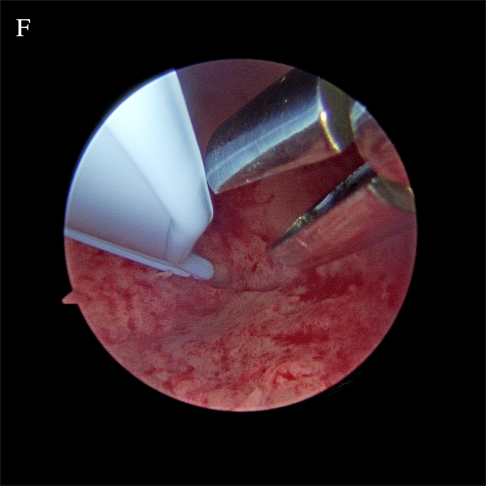


**Supplementary Figure S2. Hysteroscopic confirmation of optimal intrauterine stent positioning.**

Panels A–B show intrauterine stent placement under hysteroscopic guidance. Panel C demonstrates full contact of the stent base with the uterine fundus. Panels D–F illustrate assessment of lateral positioning of the stent within the uterine cavity. Optimal positioning was defined as full fundal contact with symmetrical lateral expansion, with the lateral margins positioned approximately one 5-Fr operative instrument width (≈1.5–2 mm) from the lateral uterine wall, as assessed by intraoperative hysteroscopic assessment. Fr, French gauge.


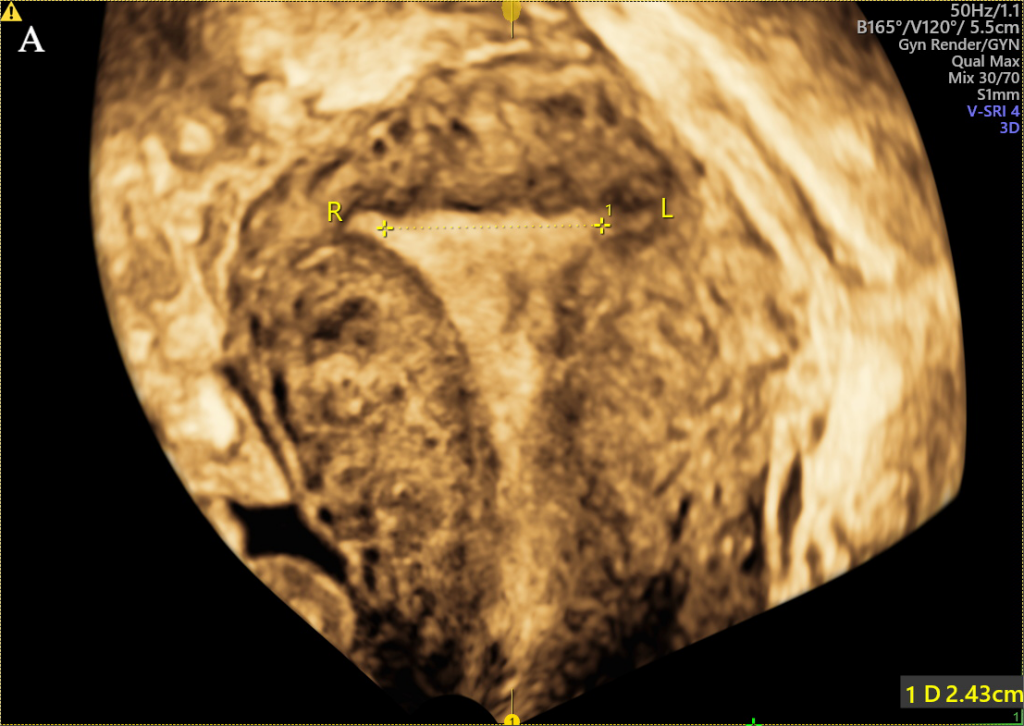

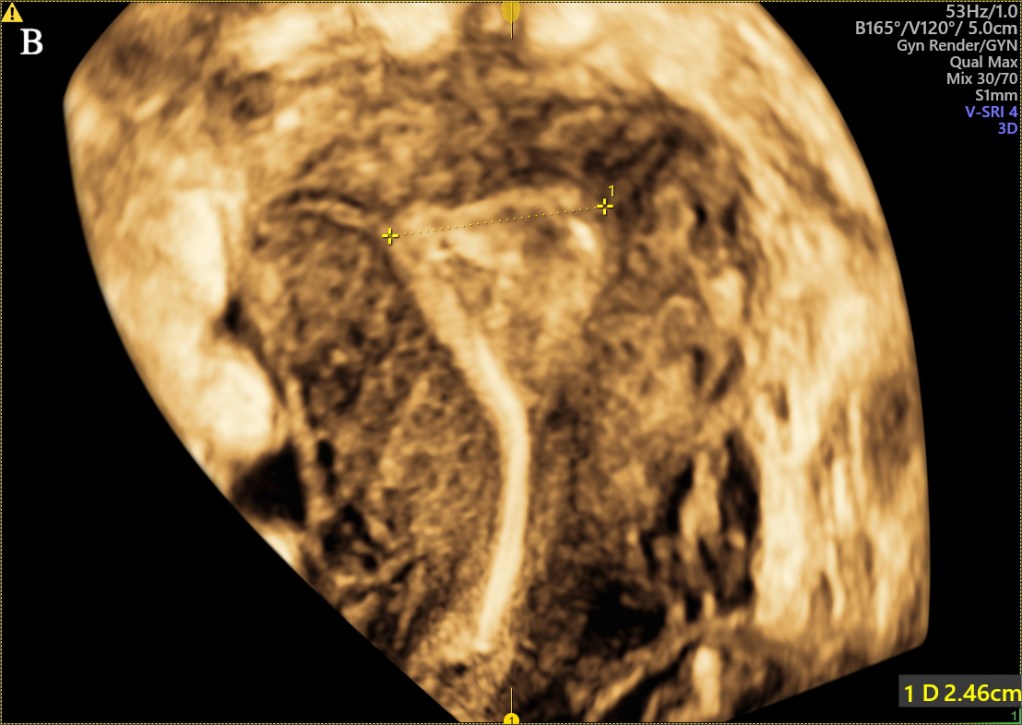


**Supplementary Figure S3. Measurement of intercornual distance (ICD) using three-dimensional transvaginal ultrasound.**

Panel A shows preoperative three-dimensional transvaginal ultrasound (3D-TVS) with measurement of intercornual distance (ICD). Panel B shows postoperative 3D-TVS following intrauterine stent placement. ICD was defined as the linear distance between the bilateral uterine cornua at the junction of the endometrial cavity and the tubal ostia. For analysis, the ultrasound examination closest in time to the initial hysteroscopic procedure (i.e. stent placement) was selected. When an intrauterine stent was visible within the uterine cavity, measurements were based on the anatomical contour of the uterine cavity rather than the stent margins to minimize device-related measurement bias. 3D-TVS, three-dimensional transvaginal ultrasound; ICD, intercornual distance.

**Supplementary Figure S4. Covariate balance before and after weighting and matching between the ≤ XS and > XS groups.**

Panels A and B show covariate balance after inverse probability of treatment weighting (IPTW) using truncation at the P1–P99 and P2.5–P97.5 percentiles, respectively. Panel C shows covariate balance after 1:2 nearest-neighbor propensity score matching (PSM). Absolute standardized mean differences (SMDs) for baseline demographic and gynecological characteristics are presented before and after adjustment. After weighting or matching, most covariates were well balanced between groups, with SMDs < 0.10. IPTW, inverse probability of treatment weighting; PSM, propensity score matching; SMD, standardized mean difference.

**Supplementary Figure S5. Kaplan–Meier analysis of time to clinical pregnancy within one year of the final hysteroscopy according to stent-size classification (≤** **XS vs > XS).**

Time-to-pregnancy analyses were performed in 608 women with documented mode of conception. The cumulative probability of clinical pregnancy within one year of the final hysteroscopy was significantly lower in the ≤ XS group than in the > XS group, as assessed by the Kaplan–Meier method and compared using the log-rank test (P = 0.002). KM, Kaplan–Meier.

**Supplementary Figure S6. Receiver operating characteristic (ROC) curves comparing predictive models for clinical pregnancy within one year of the final hysteroscopy.**

ROC curves are shown for the baseline model (including demographic, reproductive, and surgical factors), and for models additionally incorporating postoperative ultrasound-derived intercornual distance (ICD, continuous variable; denoted as US-derived ICD in the figure) or stent-size classification (≤ XS vs > XS). The addition of postoperative ultrasound-derived ICD and stent-size classification resulted in only marginal improvements in discrimination, with increases in the area under the curve (AUC) of 0.008 and 0.010, respectively. However, neither improvement was statistically significant, as assessed using the DeLong test (P > 0.05). AUC, area under the receiver operating characteristic curve; ICD, intercornual distance; ROC, receiver operating characteristic.

**Supplementary Figure S7. Calibration plots (bootstrap bias-corrected) for models predicting clinical pregnancy within one year of the final hysteroscopy.**

Calibration plots are shown for the baseline model (including demographic, reproductive, and surgical factors), and for models additionally incorporating postoperative ultrasound-derived intercornual distance (ICD, continuous variable; denoted as US-derived ICD in the figure) or stent-size classification (≤ XS vs > XS). Internal validation was performed using bootstrap resampling (B = 300). All models demonstrated acceptable calibration, with bootstrap-corrected calibration slopes ranging from 0.757 to 0.773 and Brier scores ranging from 0.202 to 0.206. After bias correction, good agreement between predicted and observed probabilities was observed across models. The dotted lines represent apparent calibration, the solid lines represent bias-corrected calibration, and the dashed diagonal line represents ideal calibration. ICD, intercornual distance.

**Supplementary Figure S8. Decision curve analysis for prediction of clinical pregnancy within one year of the final hysteroscopy.**

Decision curves are shown for the baseline model (including demographic, reproductive, and surgical factors), and for models additionally incorporating postoperative ultrasound-derived intercornual distance (ICD, continuous variable; denoted as US-derived ICD in the figure) or stent-size classification (≤ XS vs > XS). Bootstrap optimism correction was applied using 300 resamples (B = 300). Net benefit was comparable across the three models over a range of clinically relevant threshold probabilities, with no clear separation between the baseline model and models incorporating ICD or stent-size classification. The solid grey line represents the treat-all strategy, and the dashed horizontal line represents the treat-none strategy.

**Supplementary Figure S9. Random forest model for prediction of clinical pregnancy within one year of the final hysteroscopy.**

Panel A shows the receiver operating characteristic (ROC) curve of the random forest model for predicting clinical pregnancy in the test set (AUC = 0.73). Panel B shows the variable importance ranking based on the mean decrease in Gini index. As an exploratory analysis, age was the most influential predictor, with stent-size classification (≤ XS vs > XS) ranking second among the variables included in the model. AUC, area under the receiver operating characteristic curve; ROC, receiver operating characteristic; AFS, American Fertility Society; DEGO, density of endometrial glandular openings.

**Supplementary Materials and methods**

**Detailed Surgical Protocol**

All procedures were performed by the same senior surgeon with a stable surgical team in an inpatient setting. Each patient underwent two planned hysteroscopic procedures during separate hospital admissions (1–2 days per admission). The first procedure consisted of hysteroscopic adhesiolysis with intrauterine stent placement, followed by a second procedure for uterine cavity reassessment and stent removal.

In patients with preserved menstruation, surgery was scheduled during the early proliferative phase (3–7 days after the end of menstruation). In patients presenting with amenorrhea, hysteroscopic adhesiolysis was performed as early as feasible after exclusion of surgical contraindications and was not scheduled according to a specific menstrual phase. Both procedures were conducted under non-intubated intravenous anesthesia. Perioperative antibiotic prophylaxis was administered in accordance with current clinical guidelines.

A 4.9-mm outer-diameter hysteroscope with a 5-Fr working channel (KMS Medical Technology Co., Ltd., Changsha, China) was used. Routine preoperative cervical preparation was not performed. Uterine sounding and cervical dilation were not routinely performed prior to hysteroscope insertion in order to reduce the risk of uterine perforation or bleeding that could obscure the operative field (Zhou et al., 2022). Under transabdominal ultrasound guidance, the hysteroscope was advanced through the cervical canal. When adhesions or stenosis were encountered in the upper cervical canal, internal os, or lower uterine segment, blunt dissection and gradual dilation were performed as needed using 5-Fr double-joint spoon forceps to facilitate safe cavity entry.

After entering the uterine cavity, adhesions were systematically divided in an upward direction until both tubal ostia were clearly visualized and the overall uterine contour was restored. Residual scar tissue adherent to the uterine wall was further dissected using the cold scissors ploughing technique under hysteroscopic visualization (Liu et al., 2025). Satisfactory adhesiolysis was defined as re-establishment of the uterine cavity architecture with bilateral tubal ostia visible and no dense residual adhesions requiring further separation. At completion of adhesiolysis, the American Fertility Society (AFS) score (1988) and the density of endometrial glandular openings (DEGO) grade (Zhao et al., 2021) were documented. When indicated, hysteroscopic tubal catheterization and chromopertubation were performed to assess tubal patency.

Following restoration of the uterine cavity architecture, uterine cavity depth was measured using a uterine sound prior to intrauterine stent placement to ensure that the stent reached the uterine fundus. For placement, the cervix was dilated to Hegar No. 7.5–8, and the stent was inserted using a pusher. The appropriate intrauterine stent size (KMS Medical Technology Co., Ltd., Changsha, China; Supplementary Figure 1) was selected primarily according to the preoperative three-dimensional ultrasonographic intercornual distance (ICD), with intraoperative visualization of cavity width used for confirmation. Final positioning was confirmed hysteroscopically after placement. Under uterine cavity distension, optimal positioning was defined as full contact of the stent base with the uterine fundus and symmetrical lateral expansion, with each corner positioned approximately one operative instrument width (5-Fr reference, corresponding to ~1–2 mm; Supplementary Figure 2) from the lateral uterine wall. Stents not meeting these criteria were replaced, and care was taken to prevent displacement during hysteroscope withdrawal.

Second-look hysteroscopy was generally recommended after two to three natural menstrual cycles according to the routine clinical protocol at our center. However, in routine clinical practice the timing could be adjusted depending on individual clinical circumstances, including patient scheduling considerations or coordination with assisted reproductive treatment timelines (e.g. embryo transfer planning). As this was a retrospective study based on extraction of information from medical records rather than a predefined research protocol, the interval between the initial adhesiolysis and the second-look procedure varied among patients. The exact number of days between the two procedures was recorded for each individual and was subsequently included as an adjustment variable in the statistical analyses.

At the second-look procedure, the intrauterine stent—equipped with a soft retrieval tail positioned within the cervical canal—was first removed directly without cervical dilation or hysteroscopic guidance. Hysteroscopic reassessment of the uterine cavity was then performed, during which the AFS score and DEGO grade were documented. Recurrent adhesions, if present, were treated with repeat adhesiolysis. Patients were subsequently referred to the Department of Reproductive Medicine for fertility evaluation and individualized conception planning.

Perioperative complications were carefully monitored during both admissions. No major adverse events were observed, including uterine perforation, cervical laceration, fluid overload, anesthesia-related complications, or severe allergic reactions.

**Ultrasound assessment and measurement of intercornual distance (ICD)**

Three-dimensional transvaginal ultrasound (3D-TVS) examinations were retrospectively retrieved from the institutional electronic medical record system. Preoperative ultrasound assessment was routinely recommended before surgery; however, in some patients imaging had already been performed at other institutions with available ICD measurements and therefore was not repeated. For analytical consistency, the ultrasound examination closest in time to stent placement was selected. The median interval between ultrasound and surgery was 14.62 days (IQR 0.57–22.59) for preoperative examinations and 1.39 days (IQR 1.36–1.48) for postoperative examinations, as summarized in Supplementary Table 8. In routine clinical practice, early postoperative ultrasound was primarily performed to evaluate intrauterine stent position and configuration.

All ultrasound examinations were performed at the same tertiary center using Voluson E8 and Voluson E10 ultrasound systems (GE Healthcare, Chicago, IL, USA), following a standardized acquisition protocol. A three-dimensional volume dataset of the uterus was acquired using transvaginal ultrasound. ICD measurements were performed in real time on reconstructed coronal planes derived from the three-dimensional volume dataset, enabling direct visualization of uterine cavity geometry rather than relying on conventional two-dimensional views. The same measurement definition and anatomical landmarks were applied for both preoperative and postoperative ultrasound assessments.

ICD was defined as the linear distance between the bilateral uterine cornua at the junction of the uterine cavity and the tubal ostia (Supplementary Figure 3). Measurement points were determined according to fixed uterine anatomical landmarks. When an intrauterine stent was visible within the cavity, the stent margins were not used as reference points; measurements were based exclusively on the anatomical contour of the uterine cavity to minimize device-related measurement bias. Ultrasound measurement tools displayed distances to one decimal place (0.1 mm) on the imaging interface; however, in routine clinical documentation values were recorded as integers after rounding to the nearest millimeter, and all analyses in the present study used these recorded values.

In the present study, stent-size classification served as the primary exposure variable for outcome analyses. Postoperative ultrasound-derived ICD measurements were analyzed as an additional assessment to evaluate measurement consistency and agreement between the two approaches. Preoperative ultrasound measurements were incomplete and were therefore used only for exploratory analyses to assess potential changes in ICD before and after surgery.

**Supplementary References**

The American Fertility Society classifications of adnexal adhesions, distal tubal occlusion, tubal occlusion secondary to tubal ligation, tubal pregnancies, müllerian anomalies and intrauterine adhesions. *Fertil Steril* 1988;**49**:944–955.

Liu Y, Xie X, Xue P, Yuan F, Qi Y, Wang H, Wang P, Lv G, Song K, Yang Z et al. Cold scissors ploughing technique versus electrosurgical excision for hysteroscopic adhesiolysis: a multicenter randomized controlled trial. *Int J Surg* 2025;**111**:2002–2009.

Zhao X, Gao B, Yang X, Zhang A, Jamail G, Li Y, Xu D. The density of endometrial glandular openings: a novel variable to predict the live birth rate in patients with intrauterine adhesions following hysteroscopic adhesiolysis. *Hum Reprod* 2021;**36**:965–975.

Zhou Z, Zhao M, Zou L, Wu M, Xu D. Hysteroscopic dilation techniques in hysteroscopic adhesiolysis. *Zhong Nan Da Xue Xue Bao Yi Xue Ban* 2022;**47**:1586–1592.
